# Comparative effectiveness of traditional Chinese exercises for knee osteoarthritis: a systematic review and Bayesian network meta-analysis

**DOI:** 10.3389/fpubh.2025.1710610

**Published:** 2026-01-06

**Authors:** Yuan Li, Zhe Zhai, Biao Guo, Yabin Liu, Zhen An, Qun Zhai

**Affiliations:** 1 Faculty of Health Sciences and Sports, Macao Polytechnic University, Macao, China; 2 Harbin Sport University, Harbin, Heilongjiang, China; 3 Xi'an University of Posts and Telecommunications, Xi'an, Shaanxi, China; 4 Xi'an Physical Education University, Xi'an, Shaanxi, China

**Keywords:** knee osteoarthritis, traditional Chinese sports, Bayesian network meta-analysis, randomized controlled trial, middle-aged and older adult(s) populations

## Abstract

**Purpose:**

This study aims to evaluate the therapeutic effects of various traditional Chinese exercises on knee osteoarthritis (KOA) in middle-aged and older adult(s) individuals through a systematic review and Bayesian network meta-analysis.

**Methods:**

Following the PRISMA guidelines, randomized controlled trials (RCTs) were retrieved from six databases: Web of Science, PubMed, Cochrane Library, Scopus, Embase, and Google Scholar. A total of 20 RCTs involving 1,457 middle-aged and older adult(s) KOA patients were included. Interventions included Tai Chi, Baduanjin, Wuqinxi, and Yijinjing. Mean Difference (MD) was used as the effect size for continuous outcomes (WOMAC subscales, SF-36, VAS, 6MWT). A Bayesian network meta-analysis was performed, and interventions were ranked using the Surface Under the Cumulative Ranking Area (SUCRA).

**Results:**

The network meta-analysis, based on SUCRA rankings, showed: For pain relief, Yijinjing combined with electroacupuncture (WOMAC Pain SUCRA: 0.77) and Tai Chi (SUCRA: 0.72) performed best; Tai Chi was also optimal for VAS score (SUCRA: 0.97). For improving joint stiffness (WOMAC Stiffness SUCRA: 0.90) and physical function (WOMAC Function SUCRA: 0.84), Baduanjin was the most effective. Regarding quality of life, Tai Chi ranked highest for physical health (SF-36 PCS SUCRA: 0.89), while Yijinjing ranked highest for mental health (SF-36 MCS SUCRA: 0.99). Usual care (SUCRA: 0.80) and Tai Chi (SUCRA: 0.66) performed best on the 6-min walk test (6MWT).

**Conclusion:**

Tai Chi and Baduanjin appear to be highly effective non-pharmacological options for alleviating pain, improving function, and enhancing quality of life in middle-aged and older adult(s) KOA patients. These findings support individualized exercise prescriptions. However, these results should be interpreted with caution. The included studies were overwhelmingly from China, which may introduce regional bias. Future research with more diverse populations is needed to confirm these findings.

**Systematic review registration:**

https://www.crd.york.ac.uk/PROSPERO/home, identifier: CRD4202457307.

## 1 Introduction

Global population aging is a significant medical and socio-demographic challenge worldwide. The World Health Organization (WHO) defines healthy aging as the process of maintaining functional capacity to achieve health in older adults (1). Knee osteoarthritis is a chronic condition primarily affecting middle-aged and older adult(s) individuals, characterized by articular cartilage degeneration, periarticular inflammation, and progressively increasing pain and dysfunction (2).

Epidemiological studies indicate that the global prevalence of KOA is ~16.0% in individuals aged 15 and over, and 22.9% in those aged 40 and over. This translates to an estimated 654 million individuals aged 40 and above living with KOA worldwide in 2020. The incidence rate is about 203 per 10,000 person-years in individuals aged 20 and over. Notably, the prevalence and incidence rates are higher in females compared to males (3).

The economic burden of KOA is substantial. In the United States, the average annual healthcare costs for a patient with KOA are estimated to be $24,550, which is significantly higher than that for individuals without KOA. Additionally, a systematic review of cost-of-illness studies reported that the annual total costs per patient with lower-limb OA ranged from €0.7 to €12.0 thousand, with direct costs accounting for a significant portion (4).

With the aging global population, the prevalence of knee osteoarthritis is rising, becoming one of the major health issues that significantly impact quality of life (5). With the advancement of modern medical treatments, drug therapy and surgical interventions have become integral components of the conventional treatment strategy for knee osteoarthritis (6). However, these approaches are often associated with high costs and potential side effects, particularly with long-term use. Therefore, exploring safe, cost-effective, and efficient non-drug treatments, especially exercise therapy that can be self-managed by patients, has become a key focus of current research.

Middle-aged and older adult(s) individuals, who are at high risk for knee osteoarthritis, encounter more complex treatment challenges due to aging and the gradual decline in physical function. This group often has multiple coexisting chronic conditions, such as cardiovascular disease, diabetes, and osteoporosis, which limit their response to certain medical and surgical treatments and complicate the management of knee osteoarthritis. Additionally, maintaining quality of life and self-care ability is a crucial consideration in treating knee osteoarthritis in this population. Joint pain and functional limitations severely impact daily activities for many middle-aged and older adult(s) patients, exacerbating psychological stress and potentially leading to social isolation and reduced life satisfaction. Therefore, treatment choices should not only prioritize medical efficacy but also consider their impact on the patient's overall quality of life. Identifying safe, effective, and sustainable treatments for this population is essential.

Previous studies have confirmed that appropriate physical activity positively impacts the relief and management of knee osteoarthritis (7–18). Traditional Chinese exercises, such as Tai Chi, Qigong, and Baduanjin, are considered ideal for middle-aged and older adult(s) individuals due to their low intensity and holistic conditioning effects on the body. However, comparative studies on the effects of different traditional Chinese exercises on knee osteoarthritis remain limited (19–23). The most effective traditional exercises, their mechanisms of action, and the optimal methods of implementation remain unclear. Some previous meta-analyses have offered solutions, but their limitations become apparent when multiple treatments are considered. Additionally, there is a lack of systematic evaluation and comparison of the adaptability and effects of these exercises at different stages of the disease and under varying individual conditions. Specifically, previous meta-analyses have predominantly used traditional pairwise comparisons, which limits their ability to simultaneously compare multiple interventions. When needing to assess the relative effectiveness of three or more interventions, this method often fails to provide a clear ranking or a comprehensive evidence network. Furthermore, many studies have small sample sizes or have not systematically compared the differential effects of various exercise forms (such as Tai Chi vs. Baduanjin) on specific KOA symptoms (like pain, stiffness, and function). This results in a lack of direct, high-quality evidence for clinicians to support recommendations for the optimal non-pharmacological therapy. Therefore, the necessity of this study lies in filling this evidence gap. To overcome the limitations of traditional pairwise comparisons, this study adopts the Bayesian network meta-analysis (NMA) method. The suitability of this approach is its ability to construct an evidence network incorporating all relevant interventions, integrating both direct evidence (e.g., Study A vs. B) and indirect evidence (e.g., inferring A vs. B through Study A vs. C and Study B vs. C). This not only significantly enhances statistical power but also allows us to provide a unified ranking for all interventions (including Tai Chi, Baduanjin, Wuqinxi, etc.) and estimate the probability of each exercise being the best choice (24, 25). This is essential for clinical decision-making and policy development.

Therefore, this study aims to comprehensively compare and evaluate the effects of different traditional Chinese exercises on middle-aged and older adult(s) patients with knee osteoarthritis (OA) using data from randomized controlled trials (RCTs) through systematic review and Bayesian network meta-analysis. By employing this comprehensive approach, the study seeks to provide more scientific and effective exercise intervention recommendations for middle-aged and older adult(s) knee OA patients, thereby offering more precise treatment options in clinical practice and delivering more personalized solutions for their health management.

## 2 Methods

### 2.1 Registration

The study was completed in strict accordance with PRISMA (26) guidelines and it also followed the Cochrane Handbook for Systematic Reviews of interventions. The study protocol has been registered in the International Registry of prospective Systematic Reviews PROSPERO database (registration number: CRD42024573074) (27).

### 2.2 Search strategy

Six databases, including Web of Science, PubMed, Cochrane Library, Scopus, Embase, and Google Scholar, were searched from the inception of each database up to 2025, with English as the search language.

Search strategies for Web of Science, PubMed, Cochrane Library, Scopus, and Embase were developed using combinations of Medical Subject Headings (MeSH) terms, keywords, and phrases. The search terms included Knee Osteoarthritis, Randomized Controlled Trial, Tai Ji, Tai Chi, Wuqinxi, Eight-Section Brocade, Baduanjin, Yijinjing, and Traditional Chinese Exercises. A fuzzy phrase search strategy was applied in Google Scholar, retrieving the top 50 studies for each phrase, and all results were exported to EndNote20.4 software. Search formulas for Web of Science, PubMed, Cochrane Library, Scopus, and Embase are provided in Supplementary Table 1, with PubMed as an example.

### 2.3 Inclusion and exclusion criteria

#### 2.3.1 Inclusion criteria

The research questions and inclusion criteria were defined based on the PICOS principles (28). The subjects included both male and female participants, aged 45 years and above. To ensure the applicability and relevance of the study results, randomized controlled trials were selected to minimize the influence of bias and confounding factors. Seven outcome measures were included: the Western Ontario and McMaster Universities Osteoarthritis Index (WOMAC) subscales (pain, stiffness, and physical function); the visual analog scale (VAS); the short form-36 (SF-36) physical component summary (PCS) and mental component summary (MCS); and the 6-min walk test (6MWT). Only studies involving interventions using traditional Chinese exercises, such as Tai Chi, Baduanjin, Wuqinxi, and Yijinjing, were included.

#### 2.3.2 Exclusion criteria

To ensure the accuracy and high stability of the final included studies, strict exclusion criteria were applied. The exclusion criteria included: (1) non-randomized controlled trials, such as observational studies, cohort studies, cross-sectional studies, and case-control studies; (2) non-traditional Chinese exercises, such as running, swimming, and badminton; (3) systematic reviews, meta-analyses, and review articles; (4) studies that could not provide complete original data or statistical analysis results; (5) non-human studies; (6) studies involving patients without knee osteoarthritis; (7) studies with incorrect outcome indicators; (8) studies with unavailable or erroneous data, or missing literature; (9) studies involving participants under the age of 45; (10) study registration records or protocols.

### 2.4 Screening and data extraction

To reduce human bias, literature screening and data extraction were performed independently by two researchers. In the case of disagreement between the two researchers regarding a particular study, a third researcher was consulted to make the final decision after a full discussion between the two. Based on the inclusion and exclusion criteria, the researchers reviewed the title and abstract, screened relevant studies, and read the full text of the included studies to collect the following data: (1) basic information, including the country of the first author and the year of publication; (2) gender and age of the subjects; (3) study design details, such as sample size, intervention measures, intervention period, and duration; (4) relevant journal information, such as impact factors and inclusion in core databases like SCI, to ensure the quality of the research.

### 2.5 Quality assessment

The Risk of Bias (RoB) tool recommended by the Cochrane Collaboration was used to assess study quality, including randomized sequence generation, allocation concealment, blinding, data integrity, selective reporting, and bias from other sources. Each item was rated as “low risk,” “uncertain risk,” or “high risk,” based on the descriptions provided in the literature.

### 2.6 Statistical analysis

In this study, the JAGS package was used to implement the Bayesian network model, and R3.6.2 software with the GEMTC package was employed for interface connection to complete the Bayesian network meta-analysis. Model selection was based on the heterogeneity between studies, and the *I*^2^ statistic was used to measure this heterogeneity. When *I*^2^ exceeded 50%, large heterogeneity was indicated, and a random effects model was applied to capture the variability between studies. When *I*^2^ was less than or equal to 50%, low heterogeneity was observed, and the fixed effects model was considered appropriate.

The posterior distribution was sampled using Markov Chain Monte Carlo (MCMC) simulation. The process involved setting the initial value at 2.5 for four Markov chains, performing 20,000 iterations as the annealing period, and then iterating an additional 50,000 times to achieve model convergence, with a step size of 1. The deviance information criterion (DIC) was calculated to compare the goodness of fit between the consistent and inconsistent models, evaluating network consistency. Generally, a difference of DIC values >5 suggests that the inconsistent model fits better, indicating potential significant inconsistency in the network. If the difference was < 5, the consistent model was considered well-fitted, suggesting no significant difference between direct and indirect comparisons and good overall consistency.

The potential scale reduction factor (PSRF) was calculated to assess model convergence. When the PSRF value approached 1, it indicated that the model parameters had converged across the different Markov chains, implying that the difference between chains was minimal and that the posterior distribution had stabilized, with high confidence in the sampling results. After confirming convergence, posterior inference and predictive analysis were performed to ensure the robustness and accuracy of the results.

Odds ratio (OR) was used as the effect measure for binary variables, and mean difference (MD) for continuous variables. A 95% confidence interval (CI) was provided for each effect size. League tables were created to visually represent pairwise comparisons between interventions. Results were reported with 95% CIs. If the confidence interval included 0, the effect size was considered not statistically significant. If the confidence interval did not include zero, the effect size was deemed statistically significant. The ranking probability of each intervention was calculated to determine its relative effectiveness. Rankograms and surface under the cumulative ranking curve (SUCRA) were used to show the ranking and relative ranking of each intervention.

Stata 14 software was employed to create a Network Plot, visually illustrating direct comparisons between interventions and the network structure. In this plot, nodes represent interventions, and the thickness of the lines indicates the number of studies comparing two interventions. A Funnel Plot was generated to detect possible publication bias or small sample effects. The Funnel Plot assessed the robustness of the systematic review by comparing the effect size with the standard error to evaluate the symmetry of study results.

A multidimensional visualization of the essential features of the included literature was conducted using Python. The Global Distribution of Research Publications was created to visually display the geographical distribution of publications across various countries. Additionally, a variety of charts, such as 3D bar charts and 3D scatter plots, were used to present multidimensional data, including sample size, study year, and intervention type. These visualization tools facilitated a comprehensive understanding of the literature's essential features and provided intuitive data support for subsequent analyses.

## 3 Results

### 3.1 Study selection

As shown in the PRISMA flow chart, 18,396 relevant studies were retrieved from databases and registration platforms, including Web of Science (*n* = 3,683), Cochrane Library (*n* = 2,629), PubMed (*n* = 2,795), Scopus (*n* = 5,885), and Embase (*n* = 5,319). After removing 13,427 duplicate records, 6,884 records remained for the screening process.

During the screening process, studies were excluded based on the following criteria: patients not associated with knee osteoarthritis (*n* = 2,762), non-traditional Chinese sports (*n* = 2,928), non-human studies (*n* = 78), and review/meta-analysis literature (*n* = 1,023). In total, 93 articles, classified as possibly meeting the inclusion criteria, were further evaluated in full text. Additionally, 250 relevant studies were retrieved through a Google Scholar keyword search. In this screening process, studies involving patients with non-knee osteoarthritis (*n* = 63), non-traditional Chinese sports (*n* = 52), and reviews/meta-analyses (*n* = 69) were excluded. A total of 66 articles proceeded to the full-text evaluation stage.

**Figure 1:**
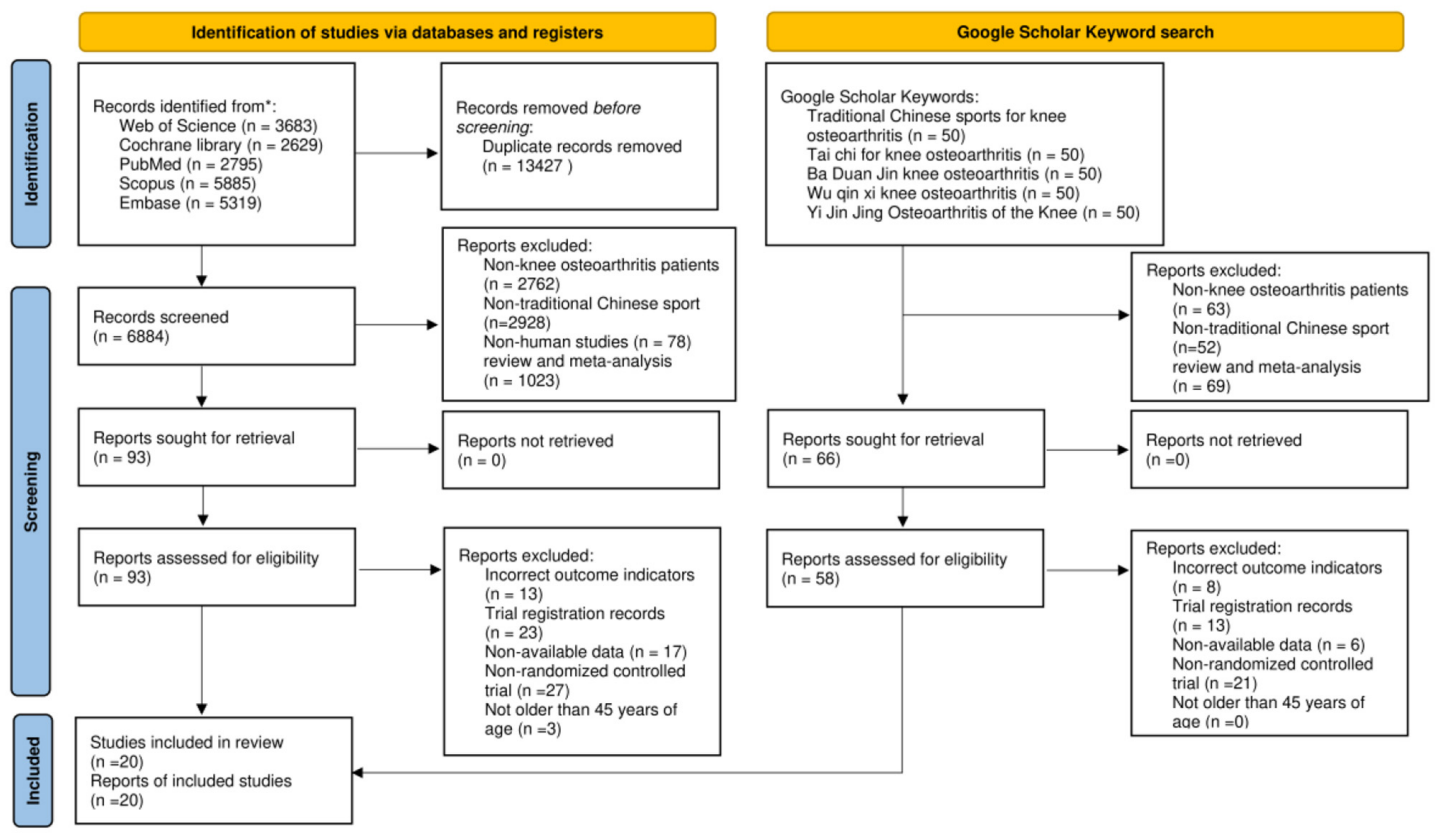
Flow chart of literature screening. *The number of records identified from each specific database.

After the full-text evaluation, studies from the database search were excluded for not meeting the outcome indicators (*n* = 13), lack of registration records (*n* = 23), absence of data (*n* = 17), being non-randomized controlled trials (*n* = 27), and involving participants under 45 years of age (*n* = 3). Similarly, articles retrieved through Google Scholar were excluded for not meeting the outcome metrics (*n* = 8), lack of registration records (*n* = 13), absence of data (*n* = 6), and being non-randomized controlled trials (*n* = 21). In total, 20 eligible randomized controlled trials (RCTs) were included for data extraction and analysis (29–48). The flow chart of study screening is presented in Figure 1.

### 3.2 Study characteristics and literature quality evaluation

#### 3.2.1 Study characteristics

A total of 20 studies were included. The subjects in these studies were all middle-aged and older adult(s) individuals, which aligns with the common characteristics of populations affected by chronic diseases such as knee osteoarthritis. Based on the pooled data, the mean age of the subjects in each study was over 45 years, with the overall mean age being 65.18 years.

Among the 20 studies, 18 were two-arm studies, and 2 were three-arm studies, involving a total of 1,457 subjects. Five different traditional Chinese exercise interventions were included. All studies were randomized controlled trials, and the 20 studies were continuous variable studies. The total duration of the experimental intervention was more than 24 h in 14 studies and less than 24 h in 6 studies. The trial intervention period was more than 12 weeks in 16 studies, and less than 12 weeks in 4 studies.

**Figure 2:**
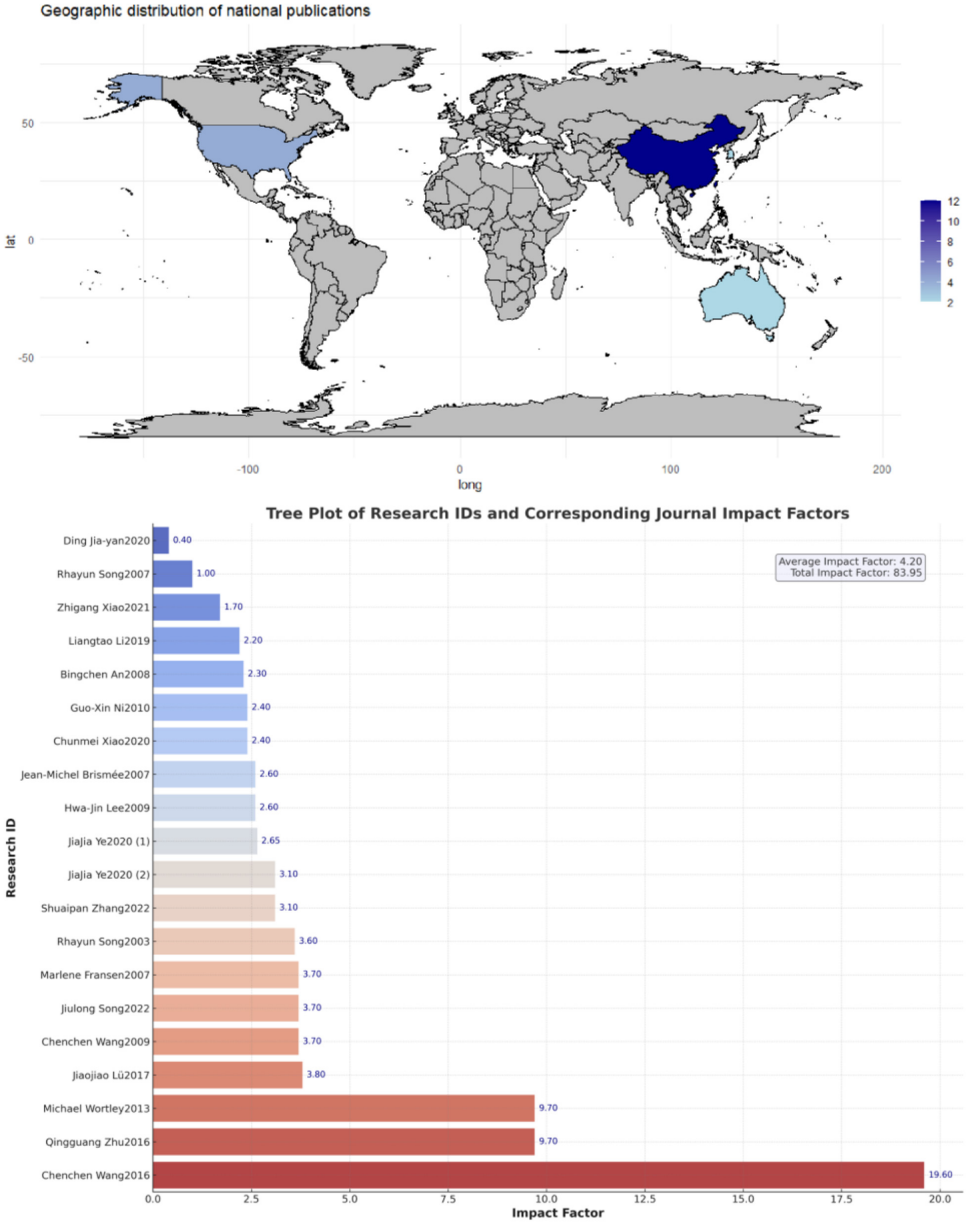
Impact factors and literature distribution map.

**Figure 3:**
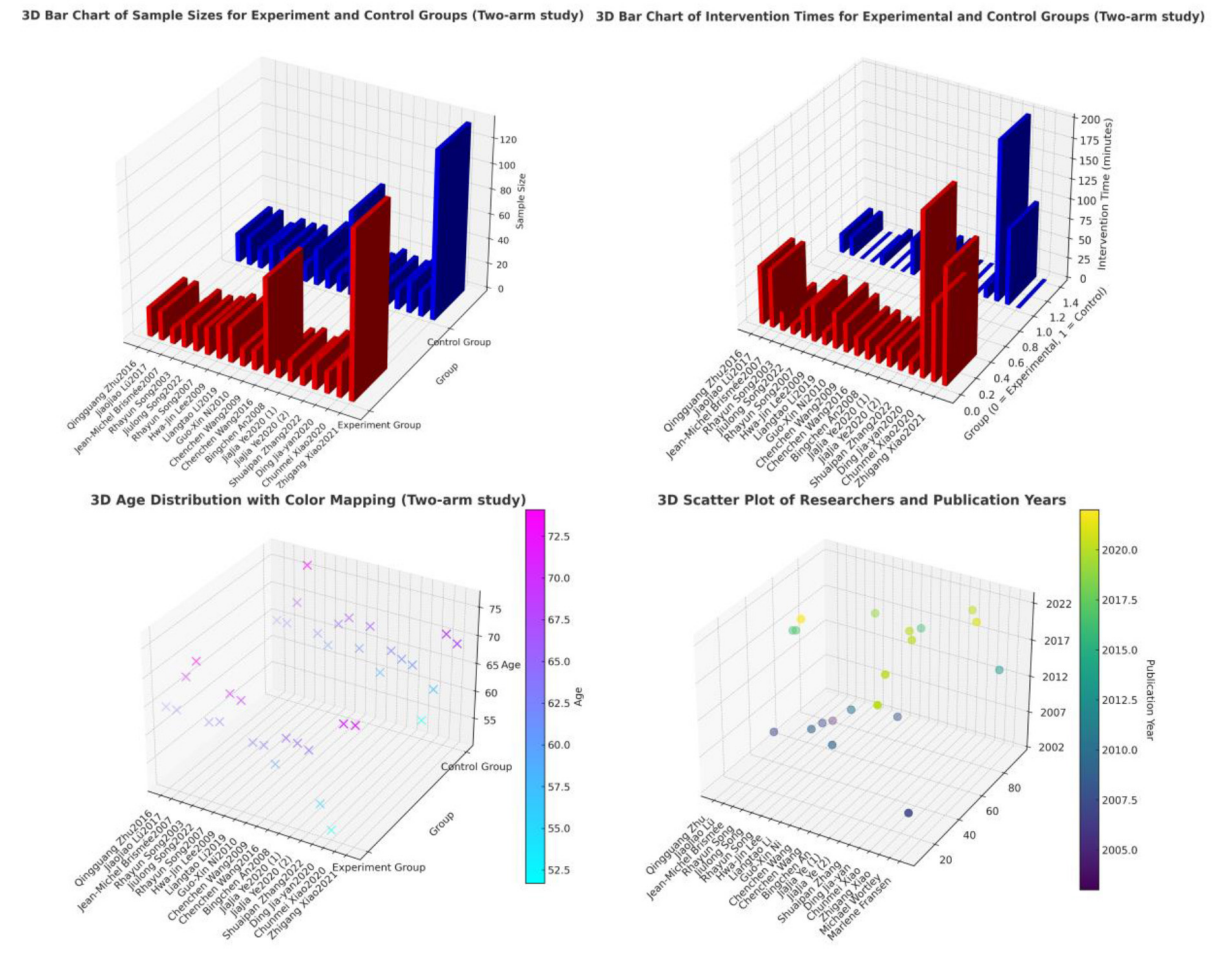
3D map of study features.

The geographical distribution of the studies was relatively concentrated, primarily in China, the United States, Australia, and South Korea. All included studies were rigorously screened and sourced from SCIE or SSCI core databases. The total impact factor of the journals was 83.95, with an average impact factor of 4.20,The outcome measures in the included studies consisted of 17 for WOMAC, 5 for VAS, 8 for SF-36 (PCS), 8 for SF-36 (MCS), and 7 for the 6-Minute Walk Test (6MWT). The main characteristics of each included study are presented in Supplementary Tables 2, 3, and Figures 2, 3.

#### 3.2.2 Evaluation of literature quality

Study quality was assessed using the cochrane risk of bias (RoB) tool recommended by the Cochrane Collaboration (49). Nineteen studies used a single-blind design, where assessors were blinded, but participants and intervention personnel were not blinded. This aspect was evaluated as having an uncertain risk. In four studies, the specific methods of allocation concealment were not described in detail, resulting in unclear risks for this part. Therefore, the evaluation of allocation concealment was considered as having an uncertain risk. One study did not employ blinding; participants and personnel were not blinded, and the report did not specify whether assessors were blinded. This was evaluated as a high risk. One study did not mention whether the assessors were blinded, and the blinded evaluation of outcome assessment was considered an uncertain risk. In two studies, the withdrawal rate was 43% in the experimental group and 39% in the control group. Although the reasons for withdrawal were primarily unrelated to health, the higher withdrawal rate may have influenced the results, and these studies were evaluated as uncertain risks due to incomplete data. The detailed results are shown in Supplementary Figures 1, 2.

### 3.3 Results of network meta-analysis

#### 3.3.1 Evidence network relationship

Each node represents a different intervention, with the size of the node reflecting the number of relevant studies. The thickness of the line indicates the strength of the comparison, and the connection between the line points signifies the presence of direct comparative evidence within the network. The area of the circle represents the sample size of the corresponding intervention study.

In the evidence network of the WOMAC subscales (Pain, Stiffness, Physical Function), the network diagram for WOMAC (pain) showed that intervention A was densely connected with several other interventions (such as B, F, G, etc.) and had more research support. The thickness of the lines indicates that intervention A has a substantial number of comparative studies with interventions such as H and I. The overall network is relatively rich. The network for WOMAC (stiffness) is simpler, with intervention A still being the core node of the research, but fewer comparisons with other interventions. The evidence network for WOMAC (physical function) is similar to that of the pain subscale, with intervention A also having strong research links with multiple interventions, especially when compared with interventions B, H, and I. The network demonstrated a broad range of studies on the improvement of knee joint function by different interventions, particularly concerning the effect on physical function limitations.

In the evidence network of SF-36 (PCS) and SF-36 (MCS), the network diagram for SF-36 (PCS) showed comparisons among multiple interventions, with intervention A being significantly supported by more research data. Thicker lines indicate frequent comparisons between intervention A and other interventions such as B, H, and G, with high evidence support. The evidence network for SF-36 (MCS) is relatively sparse, with intervention A remaining the focus of major research. Fewer and thinner lines indicate relatively few studies on the mental health dimension, although significant comparisons still exist between intervention A and other interventions such as B and F.

In the evidence network for the VAS, the network connections are relatively simple. Intervention A remains the dominant intervention, especially when compared with other interventions such as G and I.

**Figure 4:**
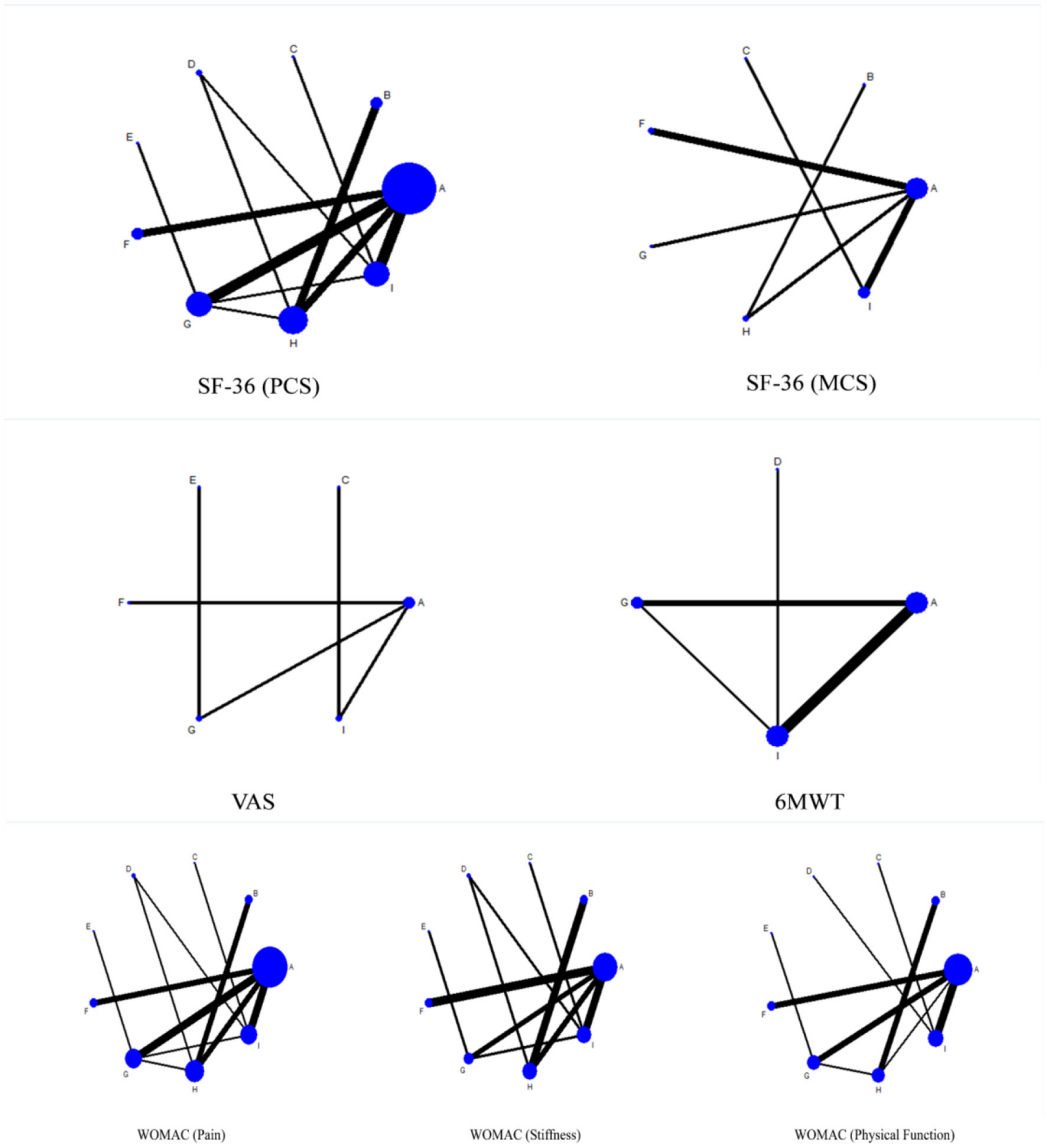
Network plots for comparisons of outcomes.

The 6-min walk test (6MWT), an indicator of functional recovery, has a relatively simple network diagram. Clear research relationships exist between intervention A and other interventions (such as G, I, etc.), but the overall network connectivity is sparse. This is shown in Figure 4.

#### 3.3.2 Consistency test, convergence diagnosis, and sensitivity analysis

To assess model convergence, the potential scale reduction factor (PSRF) was calculated. The results showed that the PSRF value for all outcome indicators was equal to 1, indicating good convergence. The deviance information criterion (DIC) was used to compare the consistency and inconsistency models. The DIC values for all outcome indicators were very close between the two models, with the absolute difference for each outcome being < 5, suggesting that the consistency model fit the data well.

For example, the DIC values for WOMAC (pain), WOMAC (stiffness), and WOMAC (Physical function) were 90.45 vs. 87.83, 71.63 vs. 71.16, and 74.29 vs. 74.34, respectively, showing minimal difference between the consistency and inconsistency models. Similarly, for SF-36 (PCS) and SF-36 (MCS), the DIC values were 30.24 vs. 31.14 and 29.55 vs. 30.79, respectively, indicating that the models were comparable in terms of fit. The VAS and 6MWT also showed small differences in DIC values (19.97 vs. 20.00, and 28.27 vs. 25.74, respectively), further supporting the consistency of the model.

These results suggest that there was no significant difference between the direct and indirect comparisons in the network, confirming the overall consistency of the model. The detailed results are shown in Supplementary Table 4, which provides the DIC values for each outcome measure in both the consistency and inconsistency models.

Additionally, a sensitivity analysis was conducted by sequentially excluding individual studies to examine the robustness of the findings. The results indicated that while some outcomes showed minor fluctuations when specific studies were excluded, the overall trends remained consistent, suggesting that the conclusions were not substantially influenced by any single study. These findings further enhance the transparency and reliability of the results.

#### 3.3.3 Evaluation of WOMAC (pain)

In the study of different traditional Chinese exercises for knee osteoarthritis in middle-aged and older adult(s) individuals, a total of 19 studies reported the WOMAC (Pain) index for 1,411 subjects. Through Bayesian network meta-analysis, comparisons between each treatment method were presented in a league table. The results showed that different traditional Chinese exercise interventions did not exhibit statistical differences, which may be attributed to the similar effects of these interventions on the WOMAC (Pain) index. The specific results are shown in Supplementary Figure 3.

The ranking probability of each intervention was calculated, and the cumulative probability ranking plot demonstrated the probability distribution of each intervention at different rankings. Intervention E (Electroacupuncture plus Yijinjing) had the highest probability of ranking first (50.96%) and performed the best, while intervention F (Health education) had a higher probability of ranking lower, indicating that its effect was relatively poor. The performance of the other interventions was more dispersed, with interventions A (Tai Chi) and B (Baduanjin) showing balanced ranking probabilities, without significant advantages or disadvantages.

**Figure 5:**
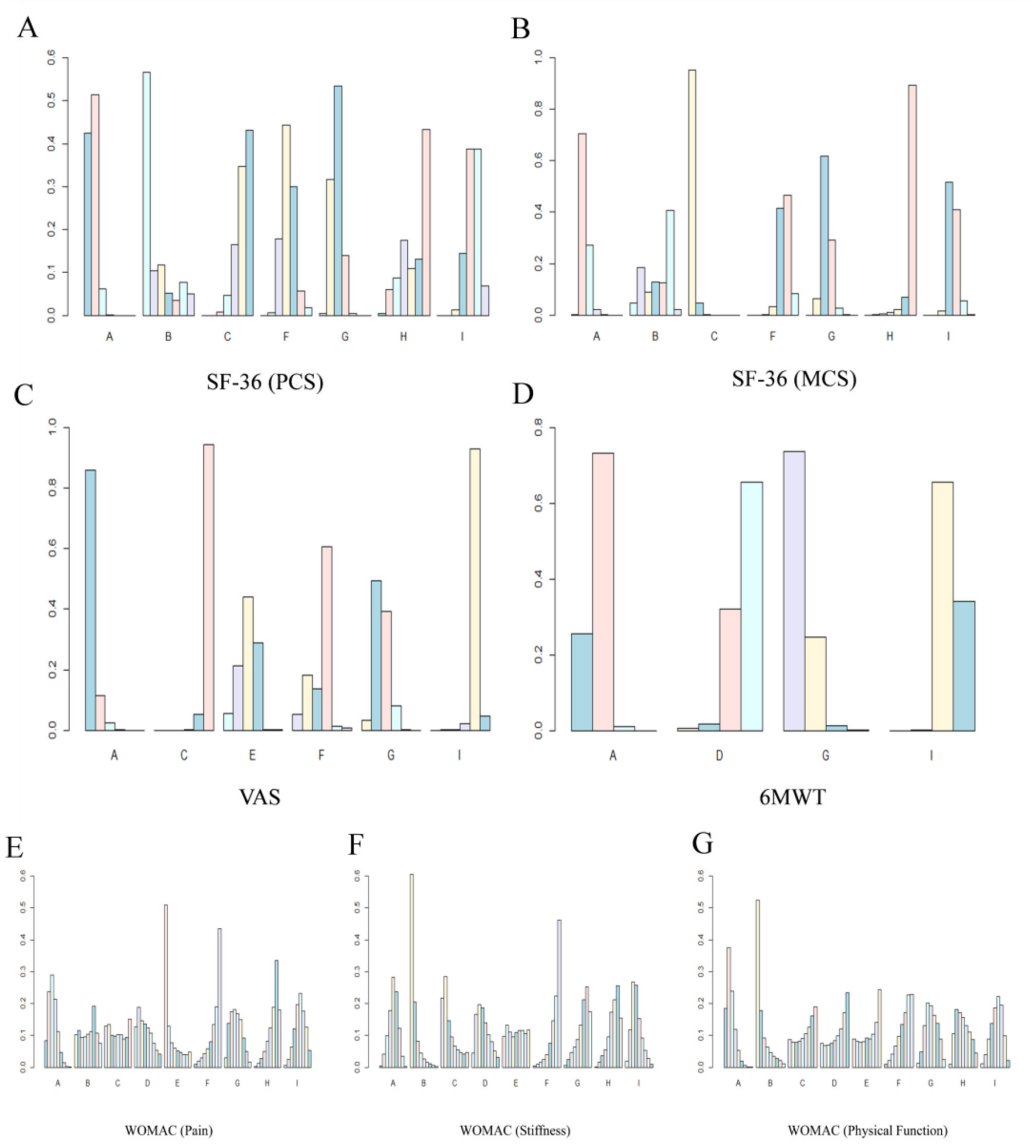
Rank probability ranking diagrams. **(A)** SF-36 (PCS); **(B)** SF-36 (MCS); **(C)** VAS; **(D)** 6MWT; **(E)** WOMAC (Pain); **(F)** WOMAC (Stiffness); **(G)** WOMAC (Physical Function). The letters on the horizontal axis represent the interventions: A, Tai Chi; B, Baduanjin; C, Yijinjing; D, Wuqinxi; E, Electroacupuncture plus Yijinjing; F, Health education; G, Usual care; H, No intervention; I, Other sports.

**Figure 6:**
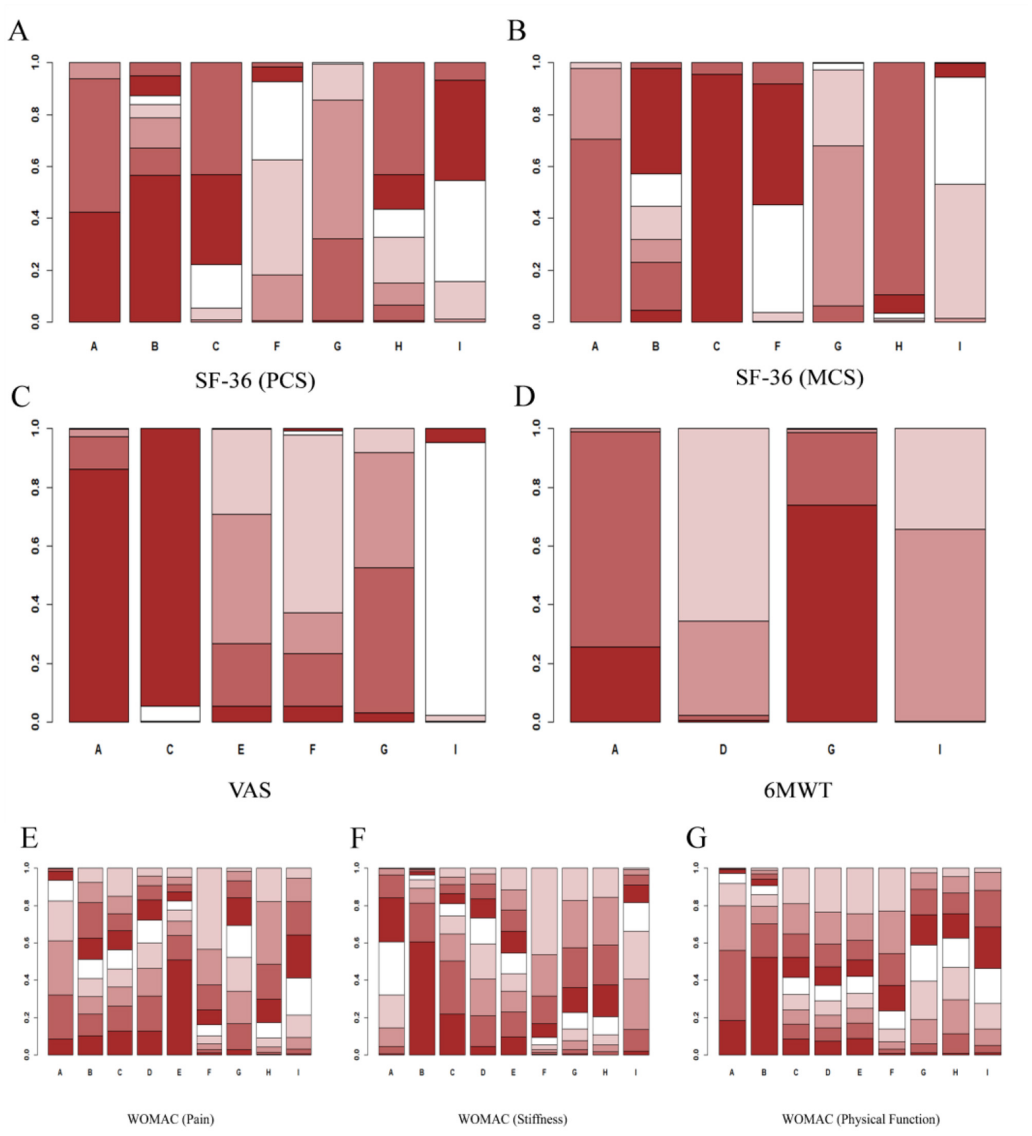
Cumulative probability ranking plots. **(A)** SF-36 (PCS); **(B)** SF-36 (MCS); **(C)** VAS; **(D)** 6MWT; **(E)** WOMAC (Pain); **(F)** WOMAC (Stiffness); **(G)** WOMAC (Physical Function). The letters on the horizontal axis represent the interventions: A, Tai Chi; B, Baduanjin; C, Yijinjing; D, Wuqinxi; E, Electroacupuncture plus Yijinjing; F, Health education; G, Usual care; H, No intervention; I, Other sports.

**Figure 7:**
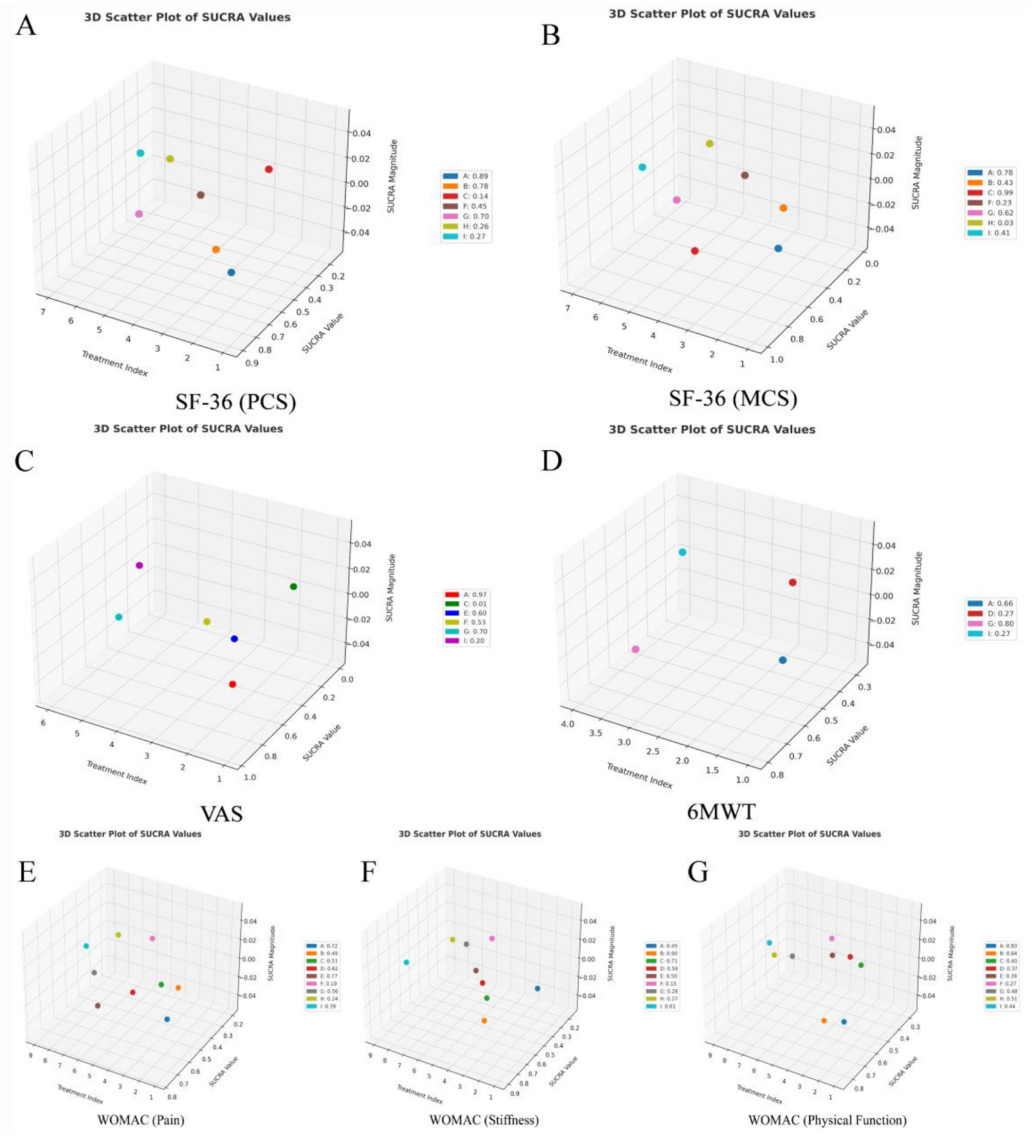
3D ranking plots of SUCRA values. **(A)** SF-36 (PCS); **(B)** SF-36 (MCS); **(C)** VAS; **(D)** 6MWT; **(E)** WOMAC (Pain); **(F)** WOMAC (Stiffness); **(G)** WOMAC (Physical Function). The letters representing interventions are: A, Tai Chi; B, Baduanjin; C, Yijinjing; D, Wuqinxi; E, Electroacupuncture plus Yijinjing; F, Health education; G, Usual care; H, No intervention; I, Other sports.

According to the calculation of the SUCRA value, the effect of each intervention was ranked as follows: Electroacupuncture plus Yijinjing (0.77) > Tai Chi (0.72) > Wuqinxi (0.62) > Usual care (0.56) > Yijinjing (0.51) > Baduanjin (0.49) > Other sports (0.39) > No intervention (0.24) > Health education (0.19). The interventions Electroacupuncture plus Yijinjing (0.77), Tai Chi (0.72), and Wuqinxi (0.62) had relatively high SUCRA values, indicating excellent treatment effects, close to the ideal optimal state. In contrast, the interventions Health education (0.19) and No intervention (0.24) had lower SUCRA values, indicating relatively poor treatment effects. These results are shown in Figures 5E, 6E, and 7E.

#### 3.3.4 WOMAC (stiffness) evaluation

In the study of different traditional Chinese exercises for knee osteoarthritis in middle-aged and older adult(s) individuals, a total of 16 studies reported the WOMAC (Stiffness) index for 1,069 subjects. The study was conducted using Bayesian network meta-analysis, and comparisons between each treatment method were shown in the league table.

It was found that intervention B (Baduanjin) compared with intervention H (No intervention) showed a statistically significant difference in the WOMAC (Stiffness) assessment, indicating that compared to No intervention, Baduanjin had the most significant effect on improving the stiffness of knee osteoarthritis in middle-aged and older adult(s) individuals. No significant differences were found for other interventions. This may be due to the similar effects of various traditional exercise interventions on improving WOMAC (Stiffness), making it difficult to observe significant differences. The specific results are shown in Supplementary Figure 3.

The ranking probability of each intervention was calculated, and the cumulative probability ranking plot demonstrated the probability distribution of each intervention at different rankings. Intervention B (Baduanjin) showed the best performance in the ranking, with a probability of 60.6% for ranking first and 20.57% for ranking second, suggesting that Baduanjin may be the most effective intervention for improving the stiffness symptoms of knee osteoarthritis. In comparison, interventions F (Health education) and G (Usual care) were ranked lower, with F having a 46.15% probability of ranking 9th, indicating that its effect may be poor. Other interventions, such as I (Other sports), had more evenly distributed rankings, suggesting a moderate effect.

According to the calculation results of the SUCRA value, the effect of each intervention was ranked as follows: Baduanjin (0.90) > Yijinjing (0.71) > Other sports (0.61) > Wuqinxi (0.59) > Electroacupuncture plus Yijinjing (0.50) > Tai Chi (0.49) > Usual care (0.28) > No intervention (0.27) > Health education (0.15). The results indicated that intervention B (Baduanjin) performed the best and was the most likely to be the optimal program for treating knee OA stiffness in middle-aged and older adult(s) patients. In contrast, interventions F (Health education) and G (Usual care) performed poorly. The remaining interventions, such as A (Tai Chi) and I (Other sports), showed moderate performance. These results are shown in Figures 5F, 6F, and 7F.

#### 3.3.5 WOMAC (physical function) evaluation

Among the studies on different traditional Chinese exercises for knee osteoarthritis in middle-aged and older adult(s) individuals, a total of 17 studies reported the WOMAC (Physical Function) index for 1,102 subjects. The study was conducted using Bayesian network meta-analysis, and comparisons between each treatment method were shown in the league table.

The results showed no statistical difference in the comparison of different traditional Chinese exercise interventions, which may be attributed to the similar effects of various traditional Chinese exercise interventions on the WOMAC (Physical Function) index. The specific results are shown in Supplementary Figure 3.

The ranking probability of each intervention was calculated, and the cumulative probability ranking plot illustrated the probability distribution of each intervention at different rankings. The results showed that the probability of intervention B (Baduanjin) achieving first place was 52.41%, with the best performance in improving the physical function of middle-aged and older adult(s) patients with knee osteoarthritis. Intervention A (Tai Chi) also performed well, with an 18.53% probability of ranking first and a 37.60% probability of ranking second. In contrast, interventions F (Health education) and G (Usual care) were mostly concentrated in the lower rankings. Intervention F (Health education) had a 22.86% probability of ranking 9th, indicating a poor effect. Other interventions, such as C (Yijinjing), D (Wuqinxi), and E (Electroacupuncture plus Yijinjing), showed more scattered ranking probabilities, suggesting that their effects were moderate.

According to the SUCRA value calculations, the effect of each intervention was ranked as follows: Baduanjin (0.84) > Tai Chi (0.80) > No intervention (0.51) > Usual care (0.48) > Other sports (0.44) > Yijinjing (0.40) > Electroacupuncture plus Yijinjing (0.39) > Wuqinxi (0.37) > Health education (0.27). Based on the calculated results, interventions Tai Chi (0.80) and Baduanjin (0.84) performed the best, indicating that they had the most significant effect in improving the physical function of middle-aged and older adult(s) patients with knee osteoarthritis. Relatively poor interventions included Health education (0.27) and Yijinjing (0.40), which showed weaker effects with low SUCRA values. Other interventions, such as Usual care (0.48), No intervention (0.51), and Other sports (0.44), had intermediate SUCRA values, suggesting that their effects were between the best and the worst. These results are shown in Figures 5G, 6G, and 7G.

#### 3.3.6 Assessment of SF-36 (PCS)

In the study of different traditional Chinese sports for knee osteoarthritis in middle-aged and older adult(s) individuals, a total of eight studies reported the SF-36 (PCS) score for 673 subjects. Bayesian network meta-analysis was conducted, and comparisons between each treatment method were shown by drawing the league table.

It was found that intervention A (Tai Chi), compared with C (Yijinjing), F (Health education), G (Usual care), and I (Other sports), intervention C (Yijinjing), compared with G (Usual care), intervention F (Health education), compared with G (Usual care), and G (Usual care), compared with I (Other sports), showed statistical differences in the SF-36 (PCS) assessment. None of the other intervention comparisons showed statistical differences. This may be attributed to the similar effects of various traditional Chinese exercise interventions in improving the SF-36 (PCS), resulting in difficulty in observing significant differences. The specific results are shown in Supplementary Figure 4.

The ranking probability of each intervention was calculated, and the cumulative probability ranking plot, together with the rank probability ranking plot, shows the probability distribution of each intervention at different rankings. The results showed that the probability of intervention B (Baduanjin) achieving first place was 56.63%, indicating the best performance. Intervention A (Tai Chi) also performed well, with probabilities of achieving first and second place being 42.43 and 51.40%, respectively. In contrast, interventions C (Yijinjing), F (Health education), and H (No intervention) had a higher probability of ranking lower, suggesting a lower effect and indicating that these interventions were less effective in improving the SF-36 (PCS) score.

According to the SUCRA value calculation, the effect of each intervention was ranked as follows: Tai Chi (0.89) > Baduanjin (0.78) > Usual care (0.70) > Health education (0.45) > Other sports (0.27) > No intervention (0.26) > Yijinjing (0.14). Based on the results, the SUCRA values of interventions A (Tai Chi) and B (Baduanjin) were 0.89 and 0.78, respectively, showing the most significant effects in improving physical health scores. In contrast, interventions C (Yijinjing) and H (No intervention) had lower SUCRA values of 0.14 and 0.26, respectively, indicating their relatively poor effectiveness under this metric. Other interventions, such as Health education (0.45) and Usual care (0.70), had moderate SUCRA values, suggesting their effects were between excellent and poor. These results are shown in Figures 5A, 6A, and 7A.

#### 3.3.7 Assessment of SF-36 (MCS)

In studies of different traditional Chinese sports for knee osteoarthritis in middle-aged and older adult(s) individuals, a total of eight studies reported the SF-36 (MCS) score for 673 subjects. Bayesian network meta-analysis was conducted, and the comparison between each treatment method was shown by drawing a league table.

It was found that intervention A (Tai Chi), compared with C (Yijinjing), F (Health education), H (No intervention), and I (Other sports), intervention B (Yijinjing) compared with H (No intervention), intervention C (Yijinjing) compared with F (Health education), G (Usual care), H (No intervention), and I (Other sports), intervention F (Health education) compared with G (Usual care), and intervention G (Usual care) compared with H (No intervention) showed statistical differences in SF-36 (MCS) assessment. Other intervention comparisons did not show statistical differences. This may be due to the similar effects of various traditional Chinese exercise interventions in improving SF-36 (MCS), which made it difficult to observe significant differences. The specific results are shown in Supplementary Figure 4.

The ranking probability of each intervention was calculated, and the cumulative probability ranking plot, together with the rank probability ranking plot, shows the probability distribution of each intervention at different rankings. The results showed that intervention C (Yijinjing) achieved first place with a probability of 95.40%, indicating that it was the most effective in improving the physical health score. The probability of intervention H (No intervention) obtaining seventh place was 89.44%, indicating that its effect was the least. Intervention I (Other sports) had a high probability of finishing fourth and fifth, with probabilities of 51.69% and 41.03%, respectively, indicating its relatively moderate effect.

According to the results of the SUCRA value, the effect of each intervention was ranked as follows: Yijinjing (0.99) > Tai Chi (0.78) > Usual care (0.62) > Baduanjin (0.43) > Other sports (0.41) > Health education (0.23) > No intervention (0.03). Based on the results, intervention C (Yijinjing) had the best performance, with a SUCRA value of 0.99, the highest among all interventions. Second, interventions Tai Chi (0.78) and Usual care (0.62) also performed well, indicating their significant effect in improving this indicator. In contrast, the No intervention group showed the least effectiveness with a SUCRA value of 0.03. Other interventions such as Baduanjin (0.43), Other sports (0.41), and Health education (0.23) had intermediate effects and average performance. These results are shown in Figures 5B, 6B, and 7B.

#### 3.3.8 Evaluation of VAS

In the study of different traditional Chinese sports for knee osteoarthritis in middle-aged and older adult(s) individuals, a total of five studies reported the VAS index for 391 subjects. Bayesian network meta-analysis was conducted, and the comparisons between each treatment method were presented through a league table.

The results indicated that significant differences were observed in the VAS assessment for the following comparisons: intervention A (Tai Chi) vs. C (Yijinjing) and I (Other sports), intervention C (Yijinjing) vs. E (Electroacupuncture plus Yijinjing), F (Health education), G (Usual care) vs. intervention E (Electroacupuncture plus Yijinjing), and intervention F (Health education) vs. I (Other sports), as well as intervention G (Usual care) vs. I (Other sports). However, no significant differences were found in other intervention comparisons. This may be due to the similar effects of various traditional Chinese exercise interventions on the VAS index, making it challenging to detect statistically significant differences. The specific results are shown in Supplementary Figure 4.

The ranking probability of each intervention was calculated, and the cumulative probability ranking plot, along with the rank probability ranking plot, illustrates the probability distribution of each intervention at different rankings. The results revealed that intervention A (Tai Chi) achieved first place with an 85.99% probability, indicating that it was the most effective in reducing pain. In contrast, intervention C (Yijinjing) was ranked 6th with a 94.4% probability, signifying it was the least effective. Other interventions, such as E (Electroacupuncture plus Yijinjing) and F (Health education), had high probabilities of intermediate rankings, indicating that their effects were between excellent and poor.

Based on the calculation of SUCRA values, the effects of each intervention were ranked as follows: Tai Chi (0.97) > Usual care (0.70) > Electroacupuncture plus Yijinjing (0.60) > Health education (0.53) > Other sports (0.20) > Yijinjing (0.01). The results show that the SUCRA value for Tai Chi was 0.97, indicating it was the most effective in reducing pain. In contrast, the SUCRA value for Yijinjing was only 0.01, reflecting that it was the least effective and had minimal impact on pain reduction. Other interventions, such as Usual care (0.70), Electroacupuncture plus Yijinjing (0.60), and Health education (0.53), performed moderately. However, the SUCRA value for Other sports was 0.20, suggesting a weaker effect. This may be attributed to the fact that middle-aged and older adult(s) patients with knee osteoarthritis are more accustomed to low-impact, slow exercises, whereas “Other sports” may involve more intense movements or higher frequencies, leading to less significant effects compared to milder traditional Chinese sports. As shown in Figures 5C, 6C, and 7C.

#### 3.3.9 Evaluation of 6MWT

In the study of different traditional Chinese sports for knee osteoarthritis in middle-aged and older adult(s) individuals, a total of seven studies reported the 6MWT indicators for 524 subjects. Bayesian network meta-analysis was performed, and the comparisons between each treatment method were illustrated through a league table.

The results revealed significant differences in the 6MWT assessment for the following comparisons: intervention A (Tai Chi) vs. D (Wuqinxi) and I (other sports), intervention D (Wuqinxi) vs. G (usual care), and intervention G (usual care) vs. I (other sports). However, no statistical differences were found in other intervention comparisons. This may be due to the similar effects of various traditional Chinese exercise interventions on the 6MWT, which made it difficult to detect significant differences. The specific results are shown in Supplementary Figure 4.

The ranking probability for each intervention was calculated, and the cumulative probability ranking plot, along with the rank probability ranking plot, illustrates the probability distribution of each intervention at different rankings. The results showed that intervention G (usual care) exhibited significant superiority, with a 73.78% probability of ranking first, indicating its most substantial efficacy in improving the 6MWT. The probability of intervention A (Tai Chi) securing second place was 73.34%, with a 25.62% probability of ranking first, signifying that its effects were substantial in most cases. In contrast, interventions D (Wuqinxi) and I (Other sports) were less effective, suggesting more modest efficacy under this metric.

Based on the calculation of SUCRA values, the effects of the interventions were ranked as follows: Usual care (0.80) > Tai Chi (0.66) > Wuqinxi (0.27) > Other sports (0.27). These results indicate that intervention G (Usual care) performed best in the 6MWT, being the most effective of all interventions. Intervention A (Tai Chi) also performed well, ranking second, while interventions D (Wuqinxi) and I (Other sports) showed relatively weak effects. As shown in Figures 5D, 6D, and 7D.

#### 3.3.10 Assessment of publication bias

To assess potential publication bias in the included studies, funnel plots were used for analysis of studies with different outcome measures. Funnel plots are a common method for detecting publication bias by examining the relationship between effect size and standard error. Ideally, scatter points should be symmetrically distributed on both sides of the funnel, indicating no publication bias. Asymmetry in the funnel plot may suggest the presence of publication bias.

The funnel plot for WOMAC (pain) showed that most scatter points were concentrated in the central region, presenting some symmetry. However, a few points were distributed in the lower-left quadrant, indicating a possible degree of heterogeneity. In the funnel plot for WOMAC (stiffness), the distribution of scatter points exhibited clear asymmetry, especially toward negative effect sizes, suggesting that studies with negative effect sizes might be underreported or biased during publication.

The funnel plot for WOMAC (physical function) displayed a certain degree of symmetry, though fewer points were found on the right side. This could indicate that some studies were not fully published, suggesting the potential for publication bias. Conversely, the funnel plot for SF-36 (PCS) index showed good symmetry, with scatter points evenly distributed around the central region. The balance of distribution on both sides of the funnel indicated no significant publication bias, suggesting that the study results under the SF-36 (PCS) index are reliable.

The funnel plot for SF-36 (MCS) showed a relatively uniform distribution of scatter points, with slight clustering on the left side. However, these scatter points were concentrated around effect sizes close to zero, suggesting a low likelihood of publication bias and reinforcing the credibility of the results.

The symmetry of the VAS funnel plot was also good, indicating that the VAS index did not exhibit significant publication bias, and the study results were reliable. The funnel plot for 6MWT showed a more scattered distribution, particularly in regions with large effect sizes, with some points far from the central region, resulting in asymmetry. This suggests a possibility of bias in the 6MWT results.

Although funnel plots for WOMAC (stiffness), WOMAC (physical function), and 6MWT showed preliminary signs of asymmetry, indicating potential publication bias, a thorough review of all included studies revealed no evidence of publication bias. This review considered factors such as the background, data sources, sample sizes, and other study characteristics. Funnel plots can exhibit asymmetry due to heterogeneity, data sparsity, or non-biased factors, so they alone cannot provide a definitive conclusion regarding bias. Further examination confirmed that all included studies were publicly published, with complete reporting and transparent data, and no studies with small samples or weak effects were excluded. The detailed results are shown in Supplementary Figure 5.

## 4 Discussion

Through systematic review and Bayesian network meta-analysis, this study comprehensively evaluated the effects of different traditional Chinese exercises on knee osteoarthritis in middle-aged and older adult(s) patients. The results of the Bayesian network meta-analysis revealed that Yijinjing combined with electroacupuncture and Tai Chi were particularly effective in alleviating pain in knee osteoarthritis patients, both showing high cumulative ranking probability (SUCRA) values. In terms of the WOMAC stiffness score, Baduanjin demonstrated the most significant effect compared to other interventions. Both Baduanjin and Tai Chi exhibited notable improvements in WOMAC physical function scores. For SF-36 (PCS and MCS) scores, Tai Chi and Yijinjing were found to be more effective than other traditional exercises in enhancing patients' quality of life, with Yijinjing showing a relative advantage in mental health outcomes. Regarding the VAS score, Tai Chi was the most effective intervention for reducing subjective pain perception in knee OA patients. The results of the 6MWT indicated that conventional treatment and Tai Chi exercise interventions were more beneficial in improving exercise endurance and walking ability.

This study is the first to incorporate the Google Scholar database in evaluating the effects of traditional Chinese sports on knee osteoarthritis in middle-aged and older adult(s) individuals. While Google Scholar does not offer medical subject headings (MeSH) or advanced search functions, which limits its role in systematic reviews and meta-analyses, its inclusion in this study helped broaden the search for relevant studies (50). However, some studies have shown that Google Scholar is more accurate, especially when complementing search results from other databases (51). To further enhance the credibility and reliability of the research, Google Scholar was used to search keywords, ensuring comprehensive data coverage and accuracy.

This study builds on existing research in several ways. A number of studies have explored the therapeutic effect of traditional Chinese exercise on knee osteoarthritis. For example, the study of Lauche et al. (52) identified the positive role of Tai Chi in relieving pain and dysfunction of knee osteoarthritis, and found that pain, physical function and stiffness of knee osteoarthritis patients who practiced Tai Chi were improved in a short period of time. It shows the potential of Tai Chi as a non-pharmacological intervention. Zeng et al. (23) found that Baduanjin exercise can reduce pain and improve physical activity in KOA patients. In addition, Li et al. (53) also evaluated the effect of traditional Chinese exercise on knee osteoarthritis through meta-analysis, which provided important evidence. However, most of these studies focused on a single form of exercise intervention and lacked a comprehensive analysis that systematically compared multiple traditional Chinese exercises.

This study is the to systematically evaluate the relative effects of multiple traditional Chinese physical exercises for knee osteoarthritis (KOA) using Bayesian network meta-analysis (NMA). One of the main advantages of Bayesian NMA is its ability to provide a more precise comparison of intervention effects by ranking the cumulative probability (SUCRA), which helps to identify not only the most effective interventions but also the likelihood of each intervention being the best. Compared to traditional meta-analysis, Bayesian NMA offers higher statistical power and analysis depth. Traditional meta-analysis typically focuses on direct evidence from pairwise comparisons between two interventions, which may limit its ability to comprehensively compare multiple treatments. In contrast, Bayesian NMA integrates both direct and indirect evidence from a wide range of studies, allowing for a broader and more precise assessment of the relative efficacy of different interventions. This approach significantly enhances the robustness of the findings, especially when dealing with small sample sizes or a variety of interventions, as it combines evidence from studies that may not have directly compared all the interventions of interest. Moreover, Bayesian NMA can evaluate multiple outcomes simultaneously, which is particularly useful when comparing traditional Chinese exercises that have diverse effects on various aspects of KOA, such as pain, physical function, and psychological wellbeing. This capability allows for a more holistic comparison of the interventions and provides a more comprehensive scientific basis for treatment decisions. By ranking interventions based on cumulative probabilities, Bayesian NMA not only provides a better understanding of which interventions are most effective but also offers a detailed framework for developing personalized treatment plans, helping clinicians choose the most appropriate exercise regimen based on individual patient needs and outcomes. This makes Bayesian NMA an especially powerful method for evaluating the complex and multifaceted effects of traditional Chinese exercises in KOA management (24). In contrast to previous studies (54, 55), This study presents an innovative approach by using the SF-36 Physical Health Summary (PCS) and Mental Health Summary (MCS) scores as key outcome measures for the first time. These scores offer a comprehensive assessment of the impact of traditional Chinese exercises on both physical and mental health, providing deeper insight into how such exercises improve the overall quality of life for knee osteoarthritis (KOA) patients. Specifically, these measures enable the evaluation of physical function and psychological wellbeing, thus allowing clinicians to gain a clearer understanding of how traditional Chinese exercises promote overall health, extending beyond pain relief and functional improvement.

Additionally, the inclusion of the 6-min walk test (6MWT) as an outcome measure enhances the assessment by evaluating the endurance and functional capacity of patients. This test provides further insight into the physical stamina of KOA patients, enabling a more thorough evaluation of the effects of traditional Chinese exercises on overall patient endurance. This metric bridges the gap between pain management, physical function, and endurance, offering clinicians a more comprehensive understanding of how traditional exercises contribute to improving both quality of life and physical wellbeing.

Notably, this study is the first to assess the effects of a combined traditional Chinese exercise intervention, such as Yijinjing in combination with electroacupuncture (EA), on patients with knee OA. While previous studies have focused on individual exercise interventions like Tai Chi and Baduanjin, this research introduces a novel perspective by exploring the combined use of multiple interventions.

A key advantage of the combined intervention is its ability to leverage the efficacy of individual exercises while enhancing the overall therapeutic effect through synergy. For instance, the combination of Yijinjing and electroacupuncture may improve knee pain and function via a dual mechanism of action, which includes the modulatory effect of traditional exercises on muscles and joints, and the stimulating effect of electroacupuncture on nerves and muscles. This combined approach offers a more personalized treatment strategy, particularly for patients who do not respond significantly to a single intervention.

In the network meta-analysis conducted, the combined intervention group demonstrated significantly better outcomes than the single intervention group in key measures such as pain relief and functional improvement, particularly in the SF-36 MCS (mental health) score. The combined approach notably improved the psychological wellbeing of patients compared to individual exercise interventions. This is likely because the integrated treatment modality can more effectively regulate both the physiological and psychological states of the patient.

For example, when evaluating VAS pain scores and WOMAC function scores, the combined intervention group generally ranked higher than the individual intervention group, indicating a clear advantage in pain relief and a greater impact on knee function. Although Tai Chi and Baduanjin alone demonstrated positive effects on pain relief and function, the combined intervention showed more significant benefits.

Given the multifaceted advantages of the combined intervention, particularly in improving patients' quality of life and mental health, it is recommended that future clinical practice consider adopting this comprehensive intervention approach. For middle-aged and older adult(s) patients with long-standing pain and dysfunction from knee arthritis, combined interventions may offer better treatment outcomes. Moreover, this strategy provides more flexibility for individualized treatment, allowing clinicians to tailor the intervention to the specific needs and conditions of each patient.

Compared with previous studies that only focused on a single form of exercise, this study significantly improved the breadth and depth of research. Despite the value of Goh et al. (56) and other studies in revealing the therapeutic effects of Tai Chi, these studies failed to cover a systematic comparison of other traditional Chinese exercise forms, especially lacking an analysis of the relative effectiveness of different interventions on multiple health indicators. However, Zhang et al. (57) only included eight studies in the basic research due to insufficient sample size, most of which were small sample studies and lack of large-sample RCT studies. Moreover, the literature quality was low, resulting in low statistical power and limiting the reliability and universality of its results in clinical application.

In contrast, this study provides a comprehensive and systematic comparison of the efficacy of each traditional Chinese exercise intervention through Bayesian network meta-analysis, which can more accurately help clinicians to choose personalized treatment regimens for different patients, and greatly improve the clinical practicability and academic value of this study.

At the same time, we found that the 20 randomized controlled trials (RCTS) included in this study were from four countries, and most of them (*n* = 13) were from China, indicating that Chinese scholars have made the greatest contribution to knee osteoarthritis intervention research. This may be related to the high attention paid to the health management of middle-aged and older adult(s) people in China in recent years. Traditional Chinese sports such as Tai Chi and Baduanjin are themselves part of Chinese culture (58), so China has a significant advantage in research in this field. Secondly, four studies were from the United States, two from Australia, and two from South Korea, indicating that these countries also have a certain academic influence on the research of knee osteoarthritis interventions in middle-aged and older adult(s) people, especially the research on aerobic exercise interventions is more concentrated in these countries. Due to the high level of economy and science and technology in these countries, there are more research funds to support the aging problem and chronic disease management, which may be one of the reasons for the concentration of research in these countries (59).

An interesting finding from the study was that Yijinjing combined with electroacupuncture (EA) demonstrated greater benefits in reducing pain, as measured by the WOMAC (pain) score, compared to single traditional Chinese exercise interventions. This enhanced effect may be attributed to the synergistic interaction of the multi-modal intervention.

Yijinjing is a traditional Chinese exercise that stretches and relaxes muscles and regulates qi and blood, which can improve the motor function and joint flexibility of patients (47). This exercise modality, by promoting blood circulation and enhancing muscle relaxation, may alleviate muscle stiffness and improve overall mobility. Additionally, Yijinjing may have an effect on the myofascial chain, which is integral to musculoskeletal pain and stiffness. The fascia, a connective tissue network, can become tight and contribute to pain and restricted movement when subjected to prolonged stress. By incorporating controlled stretching and gentle movements, Yijinjing may effectively reduce myofascial tension, thus improving joint flexibility and reducing pain.

As a form of acupuncture, electroacupuncture (EA) can directly regulate the nervous system, reduce inflammation, and relieve pain by stimulating acupoints with electric current. The analgesic effects of EA are likely due to the modulation of several physiological mechanisms, including the regulation of the neuroimmune axis. This mechanism is central to how EA influences pain. Electroacupuncture has been shown to activate the central nervous system (CNS) and trigger the release of neurotransmitters such as endorphins, which are known to suppress pain perception (60). Furthermore, EA has anti-inflammatory effects that help reduce the underlying inflammation often associated with chronic pain. Specifically, EA can modulate the release of pro-inflammatory cytokines such as IL-6, TNF-α, and IL-1β, which are key mediators of the inflammatory response in musculoskeletal disorders. By reducing the levels of these cytokines, EA not only alleviates pain but also contributes to improving joint function and mobility.

The combination of Yijinjing and electroacupuncture may produce a synergistic effect by regulating both the musculoskeletal and neural systems. Yijinjing works by promoting muscle relaxation and improving circulation, which could enhance the delivery of blood and nutrients to inflamed tissues, potentially reducing inflammation and improving the healing process. On the other hand, electroacupuncture directly targets the nervous system, influencing both the peripheral and central pathways involved in pain transmission. This dual action may be particularly effective in managing pain because it addresses the pain from both a physiological and neurological standpoint. Additionally, Yijinjing's regulation of qi and blood may help optimize the body's overall energy flow, contributing to a holistic pain-relieving effect.

Further understanding of the physiological mechanisms at play can be drawn from studies that have explored the role of the neuroimmune system in pain management. For instance, EA has been found to influence the autonomic nervous system, particularly by promoting parasympathetic activation. This can counteract sympathetic overactivation, which is often associated with pain and inflammation (61). Furthermore, EA may regulate neuroinflammatory pathways by modulating the interaction between the nervous and immune systems. Specifically, EA has been shown to influence the microglial cells in the spinal cord, which play a pivotal role in chronic pain and inflammation. By modulating these cells, EA can reduce the sensitization of the pain pathways, leading to long-term pain relief.

Moreover, recent studies have indicated that the combination of mechanical and neurophysiological therapies may lead to superior outcomes in pain management. The synergistic effects of Yijinjing and electroacupuncture likely arise from the complementary roles these interventions play in regulating the body's pain-processing systems. While Yijinjing improves the musculoskeletal system's flexibility and reduces tension, electroacupuncture provides a direct modulation of the neural circuits involved in pain, creating a more comprehensive approach to managing chronic pain associated with conditions like osteoarthritis.

In conclusion, the combination of Yijinjing and electroacupuncture may offer a more effective treatment strategy for managing musculoskeletal pain than either intervention alone. By working synergistically, these two therapies can target both the peripheral and central mechanisms involved in pain and inflammation, leading to better outcomes in terms of pain relief and functional improvement.

The results of this study provide a new clinical basis for the non-pharmacological treatment of patients with knee OA. In the future, these traditional Chinese exercise interventions can be applied to community-based rehabilitation programs, home care programs, and hospital rehabilitation treatment, especially for self-management of middle-aged and older adult(s) patients. With the further promotion of the concept of health management, exercise therapies such as Tai Chi and Baduanjin are expected to be included in health insurance plans and public health policies to help alleviate the burden of knee osteoarthritis on the older adult(s) group and improve their quality of life. With the popularity of telemedicine technology, these exercise interventions can also provide personalized health guidance through remote monitoring and digital health management platforms to further promote the long-term rehabilitation of patients with knee osteoarthritis.

Although this study provides innovative insights in many areas, several limitations must be acknowledged. First, the literature search may have affected the comprehensiveness of the results. Some studies did not report baseline patient characteristics, such as age and gender, in sufficient detail. Future research should systematically document these characteristics to better demonstrate the effects of interventions across different subgroups.

Additionally, the lack of detailed reporting on sex distribution in some studies may hinder the generalization of the findings. Considering the potential influence of gender on both the incidence of knee OA and the response to exercise interventions, future studies should include more comprehensive gender data to better understand gender-specific responses to exercise interventions.

Several methodological limitations are also noteworthy. While most of the included studies were conducted in China, the potential for publication bias has not been quantitatively assessed (e.g., using funnel plots). This may skew effect estimates, particularly if positive results from culturally specific interventions, such as Tai Chi, were preferentially published in regional databases. Moreover, cultural adaptation limitations may affect the transferability of findings. Exercise protocols based on Traditional Chinese Medicine principles (e.g., Qigong breathing techniques and movement philosophy) may lose therapeutic efficacy when directly applied to non-Chinese populations without proper adaptation.

Furthermore, socioeconomic factors, such as healthcare access, and culturally influenced attitudes toward exercise (e.g., the high adherence to group-based training observed in Chinese cohorts) may limit the generalizability of results to individualistic societies. As such, caution should be exercised when extrapolating the findings to diverse ethnic groups until multinational studies confirm these interventions in culturally appropriate contexts.

Second, most of the included RCT studies were from China, which may have a certain regional bias. Future studies should verify the effectiveness of these exercise interventions in more countries and regions. In addition, the sample size of some studies was small. For example, the sample size of Brismee et al. (30) was only 22, including 15 in the experimental group and seven in the control group. Studies with a small sample size may lead to reduced statistical power and increased uncertainty of the results, especially when performing multivariate analysis, which may not fully capture subtle differences in intervention effects. Therefore, future studies should pay more attention to the rational design of sample size to improve the external validity and statistical robustness of the findings. Given the potential impact of these limitations on our conclusions, future research must urgently advance in two key directions. First, there is a critical need for “large-scale, multicenter, multinational RCTs.” This is not only to increase sample size but also to test the efficacy, safety, and feasibility of these traditional exercises across different cultural, ethnic, and socioeconomic contexts, thereby truly assessing their global applicability. Second, future studies should not merely “report” gender data but should pre-specify “gender-based subgroup analyses” in their design. Considering the higher prevalence of KOA in women and potential sex-based differences in pain perception and response to exercise, such in-depth analysis is essential for achieving truly “individualized” exercise prescriptions.

## 5 Conclusion

This study conducted a systematic review and Bayesian network meta-analysis to comprehensively evaluate the effects of different traditional Chinese exercises on middle-aged and older adult(s) patients with knee osteoarthritis. The results of the study showed that exercises such as Tai Chi and Baduanjin had significant effects in relieving pain, improving joint stiffness and enhancing physical function, especially in the performance of Tai Chi and Baduanjin, which were significantly better than other interventions. These forms of exercise are not only effective, but also safe and economical, which are suitable for long-term health management of middle-aged and older adult(s) people.

## Data Availability

The raw data supporting the conclusions of this article will be made available by the authors, without undue reservation.
