# Supplementary material for: Comparative effectiveness of traditional Chinese exercises for knee osteoarthritis: a systematic review and Bayesian network meta-analysis
Source: Front Public Health. 2026 Jan 6;13:1710610. doi: 10.3389/fpubh.2025.1710610 (PMC12815747; doi:10.3389/fpubh.2025.1710610)
Supplement: Supplementary file 1 [file Table_1.docx]

**Supplementary materials（Figure）**

**Comparative Effectiveness of Traditional Chinese Exercises for Knee Osteoarthritis: A Systematic Review and Bayesian Network Meta-Analysis**

Yuan Li ^a,^ Zhe Zhai^b^*, Biao Guo^c,^ Yabin Liu ^d^, Zhen An ^a^, Qun Zhai ^a^

^a^Faculty of Health Sciences and Sports, Macao Polytechnic University, Macao SAR, 999078, China

^b^Harbin Sport University, Harbin, 150008, Heilongjiang, China

^c^Xi’an University of Posts and Telecommunications, Xi’an, 710121, Shaanxi, China

^d^Xi'an Physical Education University, Xi’an, 710068, Shaanxi, China

* Corresponding author.

E-mail address: zhaizhe@hrbipe.edu.cn


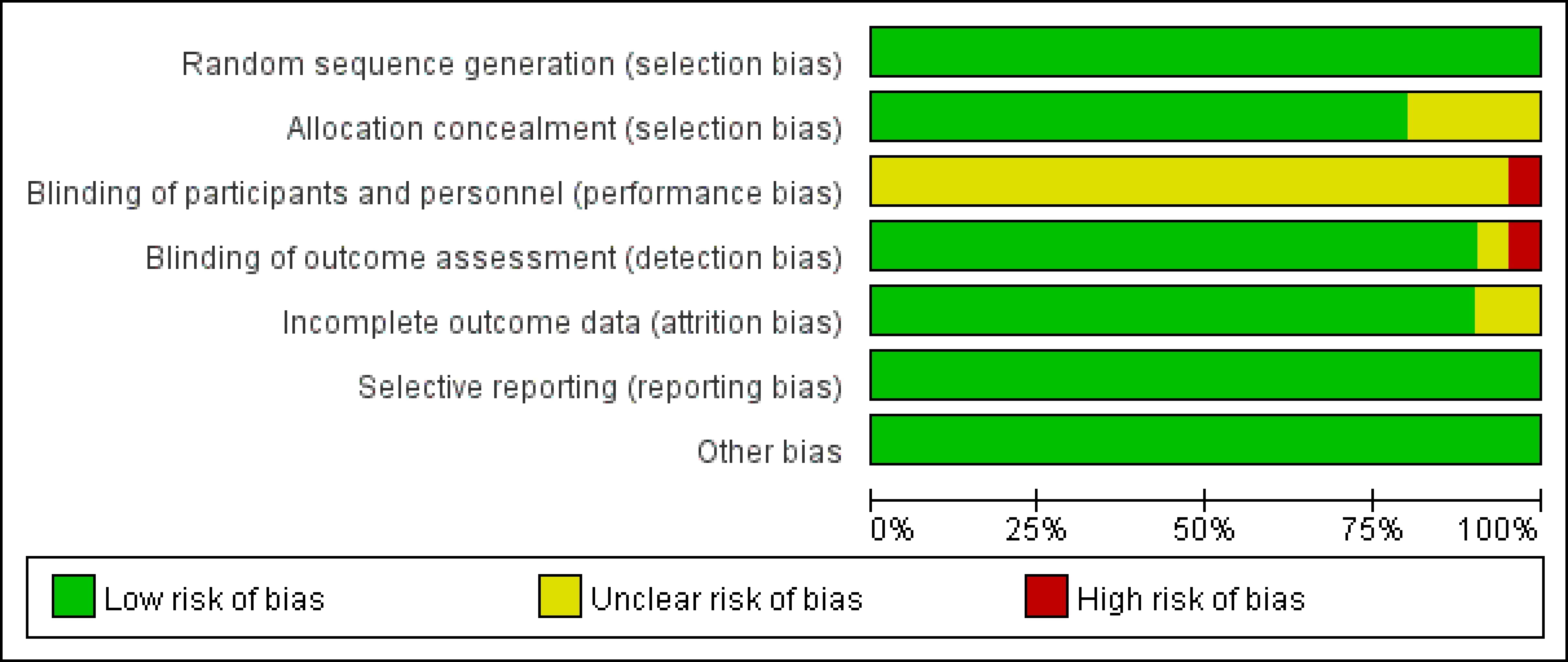


**Supplementary Fig. 1.** Risk of Bias Assessment Chart


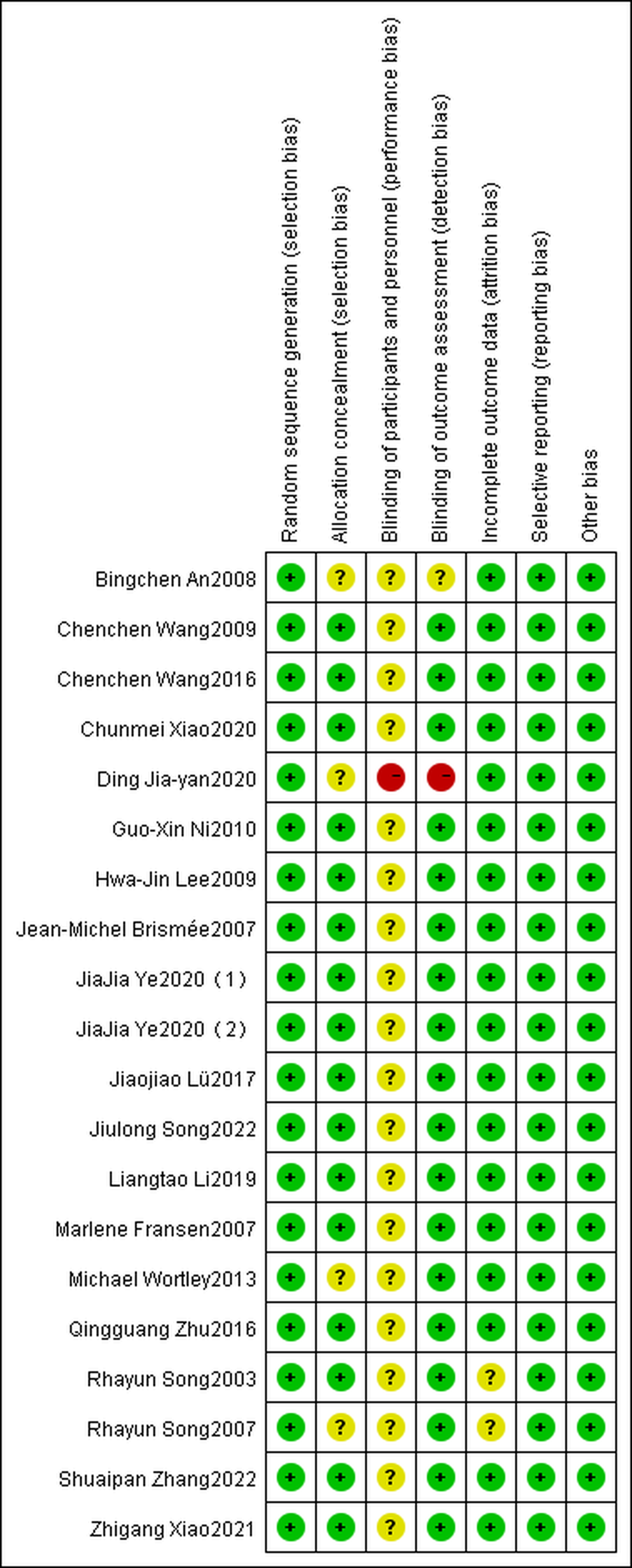


**Supplementary Fig.2.** Risk of Bias Summary

**。**


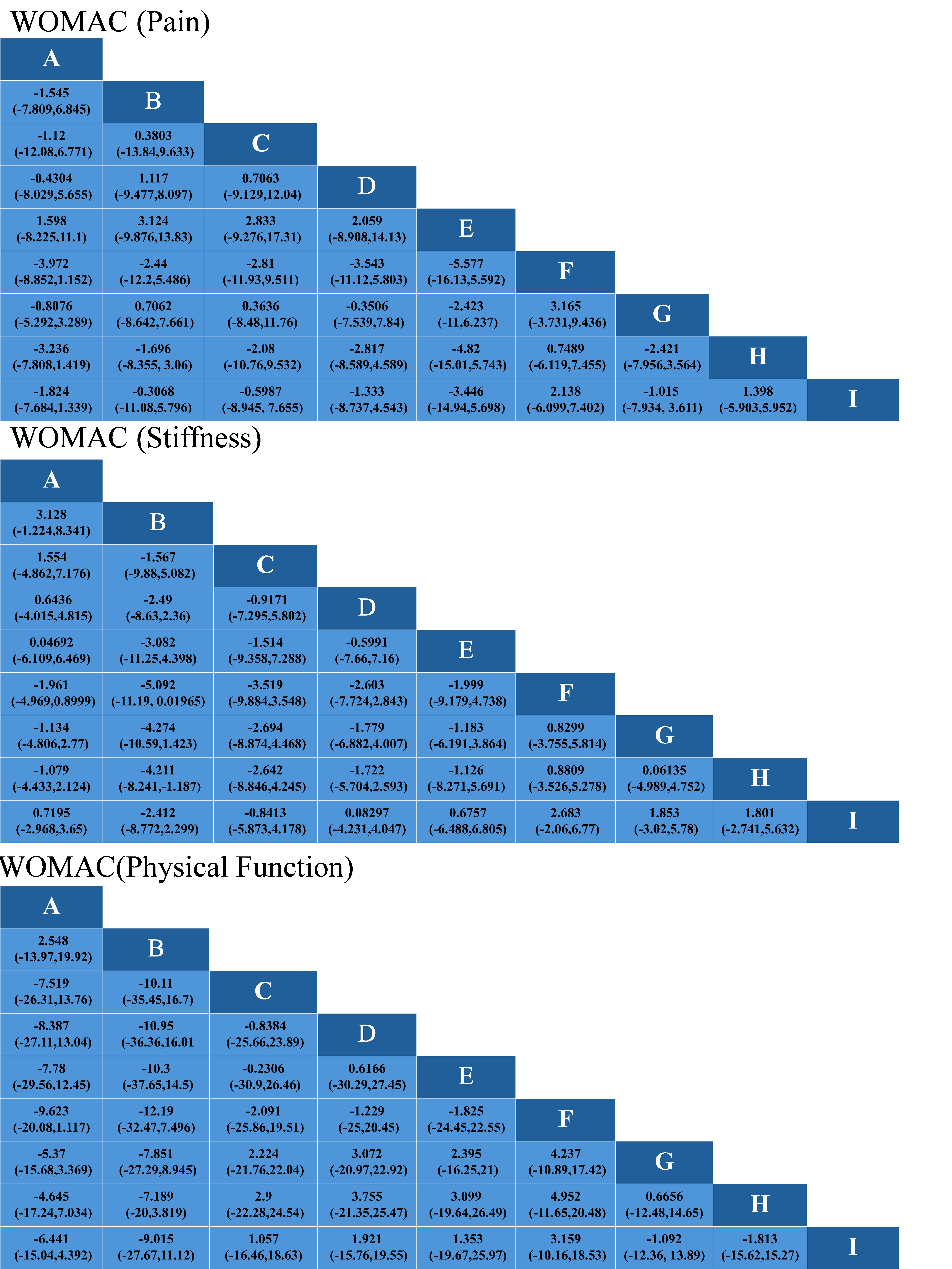


**Supplementary Fig.3.** Network Meta-Analysis Intervention Comparison Matrix


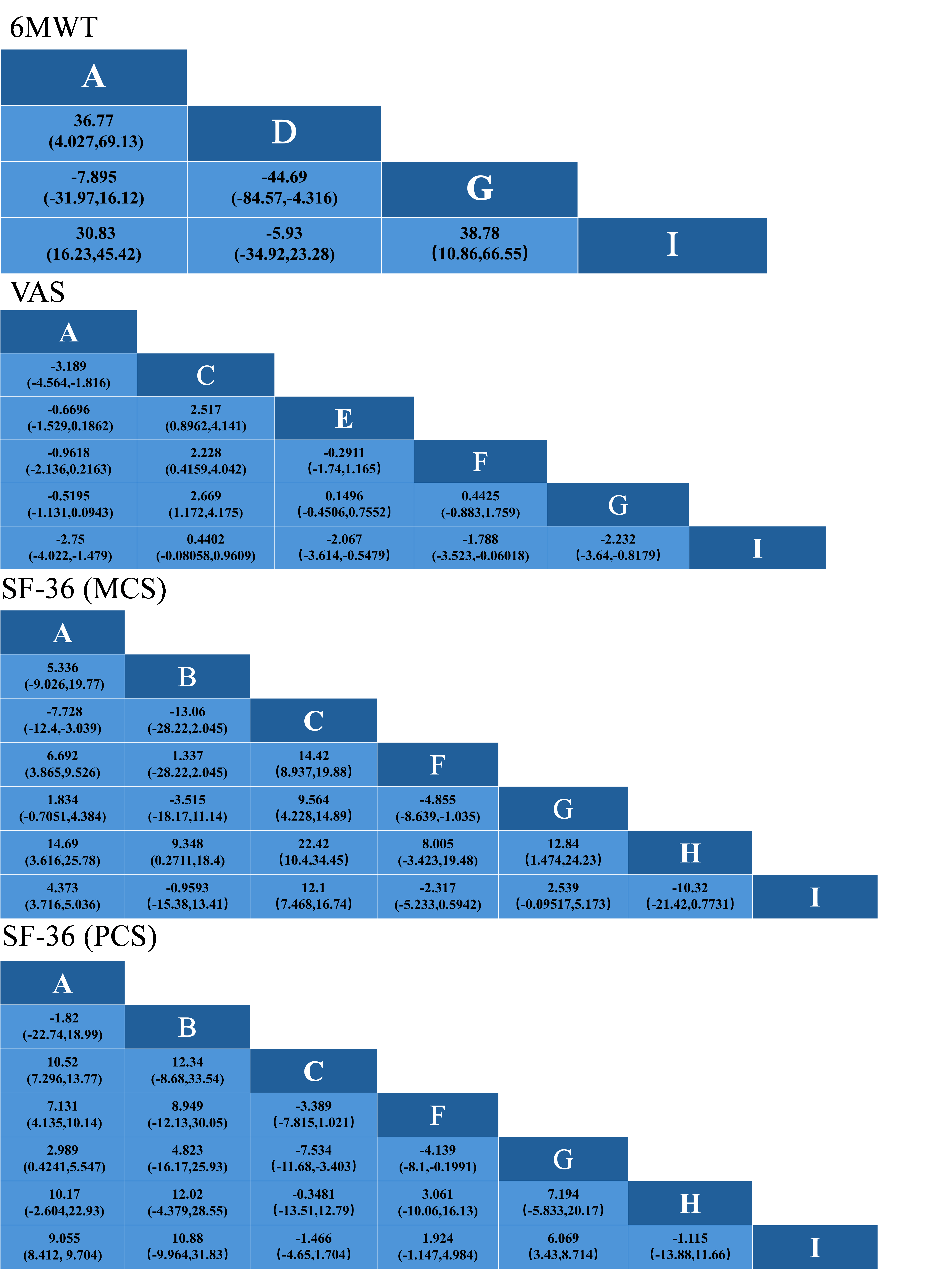


**Supplementary Fig.4.** Network Meta-Analysis Intervention Comparison Matrix


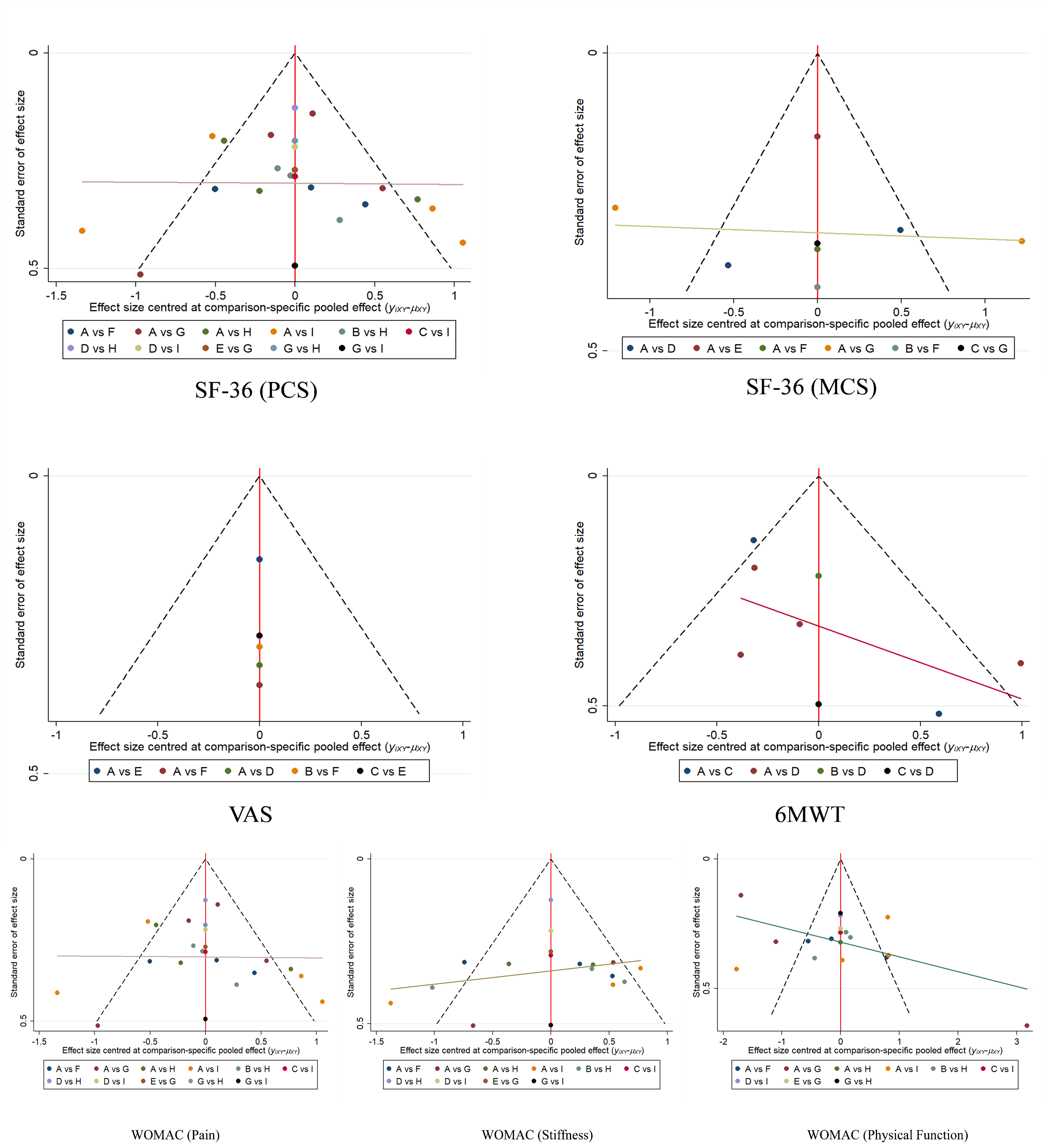


**Supplementary Fig.5.** Funnel Plot
