# Supplementary material for: Comparative effectiveness of traditional Chinese exercises for knee osteoarthritis: a systematic review and Bayesian network meta-analysis
Source: Front Public Health. 2026 Jan 6;13:1710610. doi: 10.3389/fpubh.2025.1710610 (PMC12815747; doi:10.3389/fpubh.2025.1710610)
Supplement: Supplementary file 2 [file Table_2.docx]

**Supplementary materials（Table）**

**Comparative Effectiveness of Traditional Chinese Exercises for Knee Osteoarthritis: A Systematic Review and Bayesian Network Meta-Analysis**

Yuan Li ^a,^ Zhe Zhai^b^*, Biao Guo^c,^ Yabin Liu ^d^, Zhen An ^a^, Qun Zhai ^a^

^a^Faculty of Health Sciences and Sports, Macao Polytechnic University, Macao SAR, 999078, China

^b^Harbin Sport University, Harbin, 150008, Heilongjiang, China

^c^Xi’an University of Posts and Telecommunications, Xi’an, 710121, Shaanxi, China

^d^Xi'an Physical Education University, Xi’an, 710068, Shaanxi, China

* Corresponding author.

E-mail address: zhaizhe@hrbipe.edu.cn

**Supplementary Table 1. Search strategies in Pubmed**

| **No.** | **Search content** |
| --- | --- |
| #1 | Knee Osteoarthritis[Title/Abstract] |
| #2 | "Randomized Controlled Trial" [Publication Type] |
| #3 | "Tai Ji"[MeSH Terms] |
| #4 | "tai chi"[Title/Abstract] |
| #5 | "Wuqinxi"[Title/Abstract] |
| #6 | "eight section brocade"[Title/Abstract] |
| #7 | "Baduanjin"[Title/Abstract] |
| #8 | "yi jin jing"[Title/Abstract] |
| #9 | "traditional chinese sports"[Title/Abstract] |
| #10 | ("knee osteoarthritis"[Title/Abstract] AND "Randomized Controlled Trial"[Publication Type] AND "Tai Ji"[MeSH Terms]) OR "tai chi"[Title/Abstract] OR "Wuqinxi"[Title/Abstract] OR "eight section brocade"[Title/Abstract] OR "Baduanjin"[Title/Abstract] OR "yi jin jing"[Title/Abstract] OR "traditional chinese sports"[Title/Abstract] |

**Supplementary Table 2. Basic characteristics of included studies (Two-arm study)**

| Study id | Country | Sample Size (n) | | | | | | Age (years) | | Interventions | | Duration of intervention (hours) | | Intervention Cycle (weeks) | | Intervention Frequency (times per week) | | Outcome measures ***** | Impact Factor |
| --- | --- | --- | --- | --- | --- | --- | --- | --- | --- | --- | --- | --- | --- | --- | --- | --- | --- | --- | --- |
|  |  | Experimental group | | | Control group | | | Experimental group | Control group | Experimental group | Control group | Experimental group | Control group | Experimental group | Control group | Experimental group | Control group |  |  |
|  |  | male | female | Total number | male | female | Total number |  |  |  |  |  |  |  |  |  |  |  |  |
| Qingguang Zhu2016 | China | None | 23 | 23 | None | 23 | 23 | 64.6 ± 3.4 | 64.53 ± 3.43 | Tai Chi | Health education | 72 | 24 | 24 | 24 | 3 times a week | Once a week | 1 | 9.7 |
| Jiaojiao Lü2017 | China | None | 23 | 23 | None | 23 | 23 | 64.61 ± 3.40 | 64.53 ± 3.43岁 | Tai Chi | Health education | 72 | 24 | 24 | 24 | 3 times a week | Once a week | 3 | 3.8 |
| Jean-Michel Brismée2007 | America | 3 | 12 | 22 | 4 | 15 | 19 | 70.89±9.8 | 68.8±8.9 | Tai Chi | Health education | 24 | None | 12 | 12 | 3 times a week | Twice a week | 1 2 | 2.6 |
| Rhayun Song2003 | South Korea | None | 22 | 22 | None | 21 | 21 | 74.09 ± 4.21 | 76.00 ± 5.52 | Tai Chi | Usual care | 12 | None | 12 | 12 | 3 times a week | None | 1 | 3.6 |
| Jiulong Song2022 | China | None | 20 | 20 | None | 20 | 20 | 64.15 ± 8.56 | 64.15 ± 8.56 | Tai Chi | Health education | 36 | 12 | 12 | 12 | 3 times a week | Once a week | 1 3 | 3.7 |
| Rhayun Song2007 | Australia | 0 | 22 | 22 | None | 21 | 21 | 64.8±6.0 | 62.5±5.6 | Tai Chi | No intervention | 46 | None | 12 | None | 3 times a week | None | 1 | 1 |
| Hwa-Jin Lee2009 | South Korea | 2 | 27 | 29 | 1 | 14 | 15 | 70.2±4.8 | 66.9±6.0 | Tai Chi | No intervention | 16 | None | 8 | None | Twice a week | None | 1 2 3 4 | 2.6 |
| Liangtao Li2019 | China | 26 | 28 | 54 | 24 | 29 | 53 | 69.6 ± 4.3 | 68.5 ± 3.5 | Tai Chi | Other Sports | 45 | 45 | 12 | 12 | 5 times a week | 5 times a week | 1 3 4 | 2.2 |
| Guo-Xin Ni2010 | China | None | 14 | 14 | None | 15 | 15 | 62.89 ± 2.79 | 63.47 ± 2.85 | Tai Chi | Other Sports | 36 | 18 | 24 | 24 | Two to four times a week | Once a week | 1 4 | 2.4 |
| Chenchen Wang2009 | America | 4 | 16 | 20 | 6 | 14 | 20 | 63 ± 8.1 | 68 ± 7.0 | Tai Chi | Other Sports | 24 | 24 | 12 | 12 | Twice a week | Twice a week | 1 2 3 4 | 3.7 |
| Chenchen Wang2016 | America | 31 | 75 | 106 | 30 | 68 | 98 | 60.3 ± 10.5 | 60.1 ± 10.5 | Tai Chi | Usual care | 24 | 24 | 12 | 12 | Twice a week | Twice a week | 1 2 3 4 | 19.6 |
| Bingchen An2008 | China | None | 14 | 14 | None | 14 | 14 | 65.4 ± 8.2 | 64.6 ±6.7 | Baduanjin | No intervention | 20 | None | 8 | None | 5 times a week | None | 1 3 4 | 2.3 |
| JiaJia Ye2020（1） | China | 11 | 17 | 28 | 8 | 20 | 28 | 65.11 ± 6.57 | 63.61 ± 2.63 | Baduanjin | No intervention | 24 | None | 12 | None | 3 times a week | None | 1 | 2.65 |
| JiaJia Ye2020（2） | China | 12 | 13 | 25 | 8 | 17 | 25 | 64.48 ± 7.81 | 63.08 ± 3.65 | Baduanjin | No intervention | 24 | None | 12 | None | 3 times a week | None | 1 | 3.1 |
| Shuaipan Zhang2022 | China | 4 | 21 | 25 | 9 | 16 | 25 | 55.76 ± 8.37 | 53.40 ± 10.66 | Yijinjing | Other Sports | 16 | 16 | 12 | 12 | Twice a week | Twice a week | 1 2 3 | 3.1 |
| Ding Jia-yan2020 | China | 9 | 17 | 26 | 6 | 24 | 30 | 51.7±12.9 | 59.7±10.4 | Electroacupuncture plus Yi Jin Jing | Usual care | 3 | 3 | 5 | 5 | Twice a week | Twice a week | 1 2 | 0.4 |
| Chunmei Xiao2020 | China | 17 | 28 | 45 | 20 | 20 | 40 | 70.7± 9.36 | 70.2±10.35 | Wuqinxi | Other Sports | 96 | 96 | 24 | 24 | 4 times a week | 4 times a week | 1 4 | 2.4 |
| Zhigang Xiao2021 | China | None | 132 | 132 | None | 134 | 134 | 71 ± 2.92 | 69 ± 3.72 | Wuqinxi | No intervention | 144 | None | 24 | None | 6 times a week | None | 1 | 1.7 |

Note:* Note:* Outcome measures: 1 = Western Ontario and McMaster Universities Arthritis Index(WOMAC), 2 = Visual Analogue Scale（VAS）, 3 = 36-Item Short Form Survey（**SF-36**）, and 4 = 6-Minute Walk Test（6MWT）

‘

**Supplementary Table 3.** **Basic characteristics of the included studies (Three-arm study)**

| Study id | Country | Sample Size (n) | | | | | | | | | Age (years) | | | Interventions | | | Duration of intervention (hours) | | | Intervention Cycle (weeks) | | | Intervention Frequency (times per week) | | | Outcome measures ***** | Impact Factor |
| --- | --- | --- | --- | --- | --- | --- | --- | --- | --- | --- | --- | --- | --- | --- | --- | --- | --- | --- | --- | --- | --- | --- | --- | --- | --- | --- | --- |
|  |  | Chinese traditional exercise group | | | Other Intervention Groups | | | Control group | | | Chinese traditional exercise group | Other Intervention Groups | Control group | Chinese traditional exercise group | Other Intervention Groups | Control group | Chinese traditional exercise group | Other Intervention Groups | Control group | Chinese traditional exercise group | Other Intervention Groups | Control group | Chinese traditional exercise group | Other Intervention Groups | Control group |  |  |
|  |  | male | female | Total number | male | female | Total number | male | female | Total number |  |  |  |  |  |  |  |  |  |  |  |  |  |  |  |  |  |
| Michael Wortley2013 | America | 9 | 3 | 12 | 9 | 4 | 13 | 4 | 2 | 6 | 68.1±5.3 | 69.5±6.7 | 70.5±5.0 | Tai Chi | Other Sports | Usual care | 20 | 20 | 20 | 10 | 10 | 10 | Twice a week | Twice a week | Twice a week | 1、4 | 9.7 |
| Marlene Fransen2007 | Australia | None | None | 56 | None | None | 55 | None | None | 41 | 70.8±6.3 | 70.0±6.3 | 69.6±6.1 | Tai Chi | Usual care | No intervention | 24 | 24 | 0 | 12 | 12 | 0 | Twice a week | Twice a week | Twice a week | 1 | 3.7 |

Note:* Note:* Outcome measures: 1 = Western Ontario and McMaster Universities Arthritis Index(WOMAC), 2 = Visual Analogue Scale（VAS）, 3 = 36-Item Short Form Survey（SF-36）, and 4 = 6-Minute Walk Test（6MWT）

**Supplementary Table 4. DIC results of each model**

| Outcome measures | Consistency Model | Inconsistency Model |
| --- | --- | --- |
| WOMAC (Pain) | 90.45 | 87.83 |
| WOMAC (Stiffness) | 71.63 | 71.16 |
| WOMAC(Physical Function) | 74.29 | 74.34 |
| SF-36 (PCS) | 30.24 | 31.14 |
| SF-36 (MCS) | 29.55 | 30.79 |
| VAS | 19.97 | 20.00 |
| 6MWT | 28.27 | 25.74 |

Note:WOMAC (Pain)=Western Ontario and McMaster Universities Arthritis Index (Pain);WOMAC (Stiffness)=Western Ontario and McMaster Universities Arthritis Index (Stiffness);WOMAC (Physical Function)=Western Ontario and McMaster Universities Arthritis Index (Physical Function);SF-36 (PCS)= 36-Item Short Form Survey (Physical Component Summary);SF-36 (MCS)=36-Item Short Form Survey (Mental Component Summary);VAS=Visual Analogue Scale;6MWT=6-Minute Walk Test
